# Anti-Rheumatoid Arthritis Pharmacodynamic Substances Screening of *Periploca forrestii* Schltr.: Component Analyses In Vitro and In Vivo Combined with Multi-Technical Metabolomics

**DOI:** 10.3390/ijms241813695

**Published:** 2023-09-05

**Authors:** Jia Sun, Zuying Zhou, Yang Zhou, Ting Liu, Yueting Li, Zipeng Gong, Yang Jin, Lin Zheng, Yong Huang

**Affiliations:** 1 State Key Laboratory of Functions and Applications of Medicinal Plants, Guizhou Provincial Key Laboratory of Pharmaceutics, Guizhou Medical University, Guiyang 550004, China; sunjia1202@126.com (J.S.); zu_ing@163.com (Z.Z.); zy225300@163.com (Y.Z.); t-liu@163.com (T.L.); nhwslyt@163.com (Y.L.); gzp4012607@126.com (Z.G.); jinyang4791@163.com (Y.J.); 2 School of Pharmaceutical Sciences, Guizhou Medical University, Guiyang 550004, China; 3 National Engineering Research Center of Miao′s Medicines, Guiyang 550004, China

**Keywords:** *Periploca forrestii* Schltr., rheumatoid arthritis, network pharmacology, metabolomics, pharmacodynamic substances

## Abstract

The purpose of this study was to elucidate the metabolic action patterns of *P. forrestii* against rheumatoid arthritis (RA) using metabolomics, and to obtain its potential effective substances for treating RA. First, the therapeutic effects of *P. forrestii* against RA were confirmed; second, the chemical composition of *P. forrestii* was analyzed, and 17 prototypes were absorbed into blood; subsequently, plasma metabolomics studies using UPLC-Triple-TOF-MS/MS and GC-MS were performed to disclose the metabolomics alterations in groups, which revealed 38 altered metabolites after drug intervention. These metabolites were all associated with the arthritis pathophysiology process (−log(*p*) > 1.6). Among them, sorted by variable important in projection (VIP), the metabolites affected (VIP ≥ 1.72) belonged to lipid metabolites. Finally, Pearson’s analysis between endogenous metabolites and exogenous compounds was conducted to obtain potential pharmacological substances for the *P. forrestii* treatment of RA, which showed a high correlation between five blood-absorbed components and *P. forrestii*-regulated metabolites. This information provides a basis for the selection of metabolic action modes for *P. forrestii* clinical application dosage, and potential pharmacological substances that exerted anti-RA effects of *P. forrestii* were discovered. The study provided an experimental basis for further research on pharmacoequivalence, molecular mechanism validation, and even the development of new dosage forms in the future.

## 1. Introduction

Rheumatoid arthritis (RA) is a chronic systemic autoimmune disease characterized by symmetric polyarthritis. It eventually destroys articular cartilage and bone, and causes joint deformities and dysfunction [1]. The etiology of RA is unknown. At present, it is generally believed that the pathogenesis of RA involves genetic, environmental, and endocrine factors. Several cells are implicated in the occurrence and development of RA, such as fibroblast-like synoviocytes. Inflammatory infiltration and excessive proliferation induce and aggravate the inflammatory response and release several cytokines, such as tumor necrosis factor alpha (TNF-α) and interleukin-1β (IL-1β), that stimulate cells to produce proinflammatory factors and perpetually aggravate the inflammatory response [2,3]. At present, in Western medicine, the treatment of RA is mainly aimed at relieving pain, reducing inflammation, and improving the body. Clinical Western medicine is often treated with non-steroidal anti-inflammatory drugs, glucocorticoids, and biological agents, among others. These treatments are single and are accompanied by adverse effects, such as gastrointestinal damage and abnormal liver and kidney function [4]. NSAIDs include diclofenac, ibuprofen, and others. GC mainly refers to adrenal cortical hormones such as prednisone, triamcinolone acetonide, and others. Biological agents involve TNF- α Blockers (e.g., Certolizumab), IL-1 blockers (e.g., Anakinra), anti B cell monoclonal antibodies (e.g., Rituximab, Meiluohua^®^), and others. In addition, there are also slow-acting anti rheumatic drugs (i.e., Disease Modifying Antirheumatic Drug, DMARD), including hydroxychloroquine and methotrexate [5,6].

In addition to these traditional treatments, new materials are cross-applied with the pharmaceutical field as they can increase drug stability and overall therapeutic response, and target the delivery and release of drugs. Due to its physicochemical properties and adjustability, nanosystems have many applications in diagnostic, prognostic, and therapeutic fields [7]. Currently, various nanopharmaceuticals, studied for the treatment of RA, including DEN-181, 89Zr-DFO-CZP, and pegsunercept, have entered the clinical trial stage [8]. In the treatment of RA, in addition to discovering, identifying, detecting, or quantifying the pharmacological effects and effects of different TCM compounds, there is also an urgent problem to be solved in terms of reaching the target site. In this context, nanomedicine management and its potential application in RA have become crucial. There have been many reports covering the cross-application of TCMs or TCM small molecules and nanomaterials, which is a hot spot in the development of drug delivery systems for TCM [9]. In order to improve the bioavailability, biocompatibility, pharmacokinetics, and pharmacology of anti-RA drugs, liposomes, polymeric nanoparticles, nanobodies, and polymeric micelles have been used in some studies for RA treatment [10,11].

For example, ginsenoside Rg3 completely replaces cholesterol as a liposome membrane material and can actively target the delivery of dihydroartemisinin and paclitaxel, which are used in the treatment of triple-negative breast cancer at a lower dose [12]. Curcumin is highly fat-soluble and difficult to dissolve in water. The dosage form is modified by nanotechnology, and the solubility can be significantly improved after being turned into curcumin nanoemulsion, which can increase the uptake of the drug in vivo [13]. Paeonol is a water-insoluble active ingredient in TCM with low bioavailability. After being encapsulated in poly(lactic-co-glycolic acid) (PLGA) and transformed into paeonol PLGA nanoparticles, it can improve the solubility, increase the circulation time in the body, and achieve passive targeting [14]. Glycyrrhizic acid is an acidic triterpene saponin compound. Glycyrrhizic acid forms a salt in an alkaline environment in the body to reduce its solubility (becoming an ionic state). When the nano dosage form is transformed into glycyrrhizic acid liposome, the solubility and targeting can be significantly improved [15].

Hence, there is an urgent need for alternative medicine to treat RA. In recent years, several scholars have looked to traditional Chinese medicine (TCM) to treat RA because this method has multi-component, multi-path, and multi-target effects. Therefore, the search for new drugs against RA within the compendium of traditional Chinese herbal medicine is now a research hotspot [16]. TCM treatment often classifies and differentiates RA according to its characteristics, and TCM has high safety, overall regulation, and other characteristics that, in the clinical treatment of RA, have significant advantages [17].

*Periploca forrestii* Schltr. (*P. forrestii*, Miao medicine, Chinese name: Hei-Long-Gu) is a folk remedy widely used to treat rheumatism and rheumatoid diseases and belongs to the *Periploca* genus of the *Apocynaceae* families [18]. Clinical applications of *P. forrestii* extracts and its derivatives have curative efficacy on rheumatism and rheumatoid disease [19]. According to domestic and foreign studies [20,21], *P. forrestii* contains steroids, cardiac glycosides, flavonoids, and other constituents. Pharmacologically, it is anti-inflammatory, analgesic, antirheumatic, and immunosuppressant, among other effects.

In the early stages of this research, we analyzed the chemical constituents of *P. forrestii*, and we isolated caffeoylquinic acid compounds from it, including 3-*O*-caffeoylquinic acid, 4-*O*-caffeoylquinic acid, 5-*O*-caffeoylquinic acid, and others [21]. We also extracted cardiac glycosides such as periplogenin and periplogenin A [22]. Related research showed that caffeoylquinic acid monomers significantly inhibit the proliferation of human RA fibroblast-like synoviocytes (MH7A). These compounds may also reduce MAPK signaling pathway activity by downregulating NF-κB, inhibiting downstream proinflammatory factors, and promoting an anti-arthritic effect [23]. They can also inhibit the proliferation of MH7A cells induced by TNF-α and the secretion of proinflammatory factors such as nitric oxide, Prostaglandin E2, IL-1β, and IL-6 [24].

TCM consists of various chemical components and each of these has different pharmacological effects. Moreover, there are both synergies and antagonisms among them. Therefore, it is challenging to elucidate the exact mode(s) of action of TCM [25]. Certain studies have shown that it is important to clarify the substances in TCM that have activity against RA and identify their in vivo processes and mechanisms. At present, the material basis and the pharmacodynamics of *P. forrestii* are unclear. Conventional pharmacological methods have only partially disclosed the mechanism(s) of *P. forrestii*. Therefore, the modes of action of *P. forrestii* merit further investigation.

Metabolomics is an important part of systems biology. As it is holistic, dynamic, and immediate, it is consistent with the holistic and perpetual dynamic views emphasized by TCM. Metabolomics is more frequently applied to the study of TCM. It uses modern analytical techniques, such as nuclear magnetic resonanceNMR, gas chromatography-mass spectrometry (GC-MS), liquid chromatography-mass spectrometry (LC-MS), capillary electrophoresis-mass spectrometry (CE-MS), and others, for the qualitative and quantitative detection of endogenous metabolism of small molecules in biological samples. These techniques are also implemented to investigate changes in the endogenous metabolites of organisms under physiological and pathological conditions, and before and after drug interventions. They may localize potential disease biomarkers, clarify drug mechanisms, reveal pathogenesis, and provide references for the diagnosis and treatment of disease [26]. Metabolomics was applied here to study the effects and mechanisms of *P. forrestii* in RA treatment. The output of this analysis may help to develop novel, safe, and efficacious therapies for RA based on *P. forrestii*.

In metabolomics research, a large number of metabolites are extensively simultaneously analyzed to reveal metabolome characteristics in physiological and pathological states. Changes in metabolite levels are the result of genetic or environmental factors, as well as cellular and systemic changes in disease, microbiota, drugs, toxins, and lifestyle [27]. Therefore, an analytical platform for metabolomics research should be able to simultaneously analyze hundreds of metabolites from complex biological samples and be able to monitor changes in these metabolites [28].

Chromatography coupled with mass spectrometry can be used for the accurate and reproducible analysis of hundreds to thousands of metabolites in biological fluids or tissue samples. The number of identifiable metabolites in biological fluids or tissues varies according to the chromatographic technique used (gas or liquid chromatography), and the coverage of these chromatographic techniques also varies by metabolome [29]. Therefore, some metabolites can only be analyzed by LC or GC. However, more than one metabolite or metabolome may be found to be altered in a given disease.

Therefore, in the process of disease diagnosis, GC-MS and LC-MS techniques are usually used together to identify altered metabolites in the body, and the integration of metabolomics data from multiple analytical techniques can provide more comprehensive metabolite profile information, so that the results are more perfect, and the results of different analysis techniques are mutually verified, so as to achieve the complementary advantages of the analysis platform [30].

This study aims to conduct metabolomics research using LC/GC-MS technology to evaluate the efficacy of *P. forrestii* in rescuing abnormal metabolic product levels, combined with blood component analysis to explore its anti-RA pharmacological substances. This study is the first to analyze the blood-absorbed components of *P. forrestii*, and also the first to explore the metabolic regulation mechanism of *P. forrestii* in preventing and treating RA. Therefore, the implementation of this study can fill the gaps in the corresponding fields and provide an experimental basis for further drug equivalence, molecular mechanism validation, and even the development of new dosage forms. It can also provide a basis for the selection of dosage for *P. forrestii* clinical application in terms of the metabolic mode of action and provide an experimental basis for further clinical application.

## 2. Results

### 2.1. Basic Physiological Parameters, Hematoxylin-Eosin (H&E) Staining, and Quantification of Serum RF, TNF-α, and IL-1β

**Figure 1:**
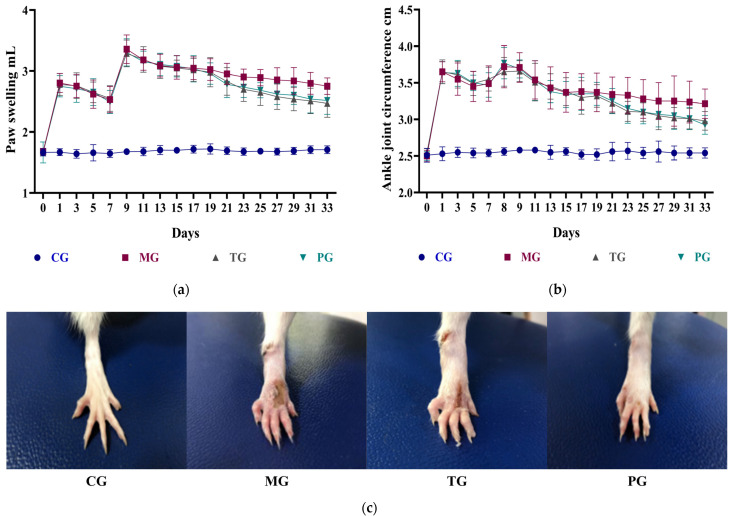

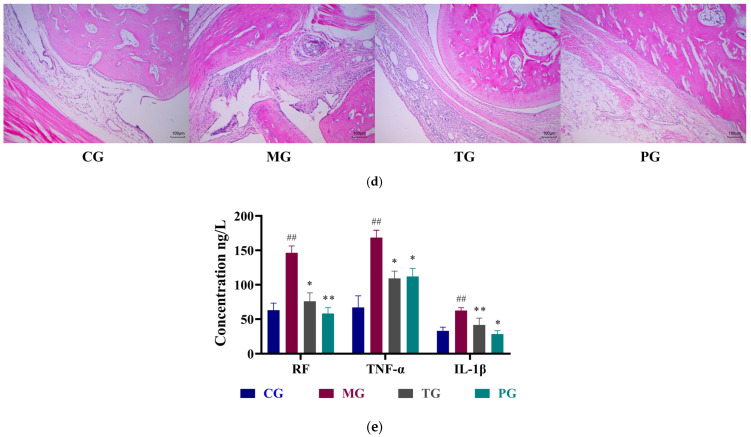
Rats’ basic physiological parameters, evaluation of AA model construction, and efficacy of the *Periploca forrestii* Schltr. extracts: (**a**) Paw swelling, *n* = 8; (**b**) Ankle joint circumference, *n* = 8; (**c**) Effects of rats in each group on paw swelling; (**d**) Hematoxylin-eosin staining and optical microscopy (100×); (**e**) Plasma levels of inflammatory factors RF, TNF-α, and IL-1β, *n* = 8, **^##^** *p* < 0.01 vs. CG; * *p* < 0.05, ** *p* < 0.01 vs. MG. Note: CG means control group; MG means model group; TG means *Periploca forrestii* Schltr. extracts treatment group; PG means positive group.

Here, we used an FCA-induced arthritis model to test the therapeutic efficacy of intragastric *P. forrestii* administration. The experimental results (Figure 1a–c) showed that during the entire experiment, the paw swelling and ankle joint circumference of the control group (CG) were virtually unchanged. The other groups presented with swelling peaked by day 3 and began to reduce between days 3 and 7. After the second immunization on day 7, the foot volumes of the model rats in each group reached their maxima by day 9 and slowly decreased between days 9 and 17. Compared with the CG group, the foot volumes and ankle joint circumferences of all other groups, except the treatment group (TG) and the positive group (PG), were significantly increased. The foot volumes and ankle joint circumferences of the TG and PG groups were significantly reduced relative to those of the model group (MG).

H&E staining and optical microscopy were used to evaluate the morphological changes in the arthritic rats (Figure 1d). The joint structure of the CG group was normal. Their synovial cells were arranged in a single layer, there was no inflammatory cell infiltration, and neovascularization was observed. Compared with the normal group, the joint structure of the MG group was severely damaged, and the synovial cells had substantially proliferated and became multilayered and disorganized. There was abundant inflammatory cell infiltration, neovascularization, sub-fiber fibrous tissue proliferation, and vascular crest formation. After treatment with *T. wilfordii* polyphenol tablets and *P. forrestii*, the lesions in the TG and PG groups were significantly reduced. These results demonstrated that the *T. wilfordii* polyphenol tablets and *P. forrestii* extracts delayed the pathological progress in the RA joints and exerted dramatic therapeutic efficacy in this model.

The proinflammatory factors RF, TNF-α, and IL-1β are released in large quantities from the joint lesions of RA patients. There, they induce an inflammatory response. This experiment measured and compared the proinflammatory factors against the CG group (Figure 1e). The plasma RF, TNF-α, and IL-1β levels were significantly higher in the MG group than the others (*p* < 0.01). Hence, the adjuvant arthritis (AA) model was successfully induced. Intervention with *T. wilfordii* polyphenol tablets and *P. forrestii* significantly (*p* < 0.05) lowered the proinflammatory cytokine concentrations in the TG and PG groups. This finding reveals that both *T. wilfordii* polyphenol tablets and *P. forrestii* have comparable and nearly equal efficacy against RA.

### 2.2. Component Analyses In Vitro and In Vivo and the Acquisition of Active Ingredient Groups

**Figure 2:**
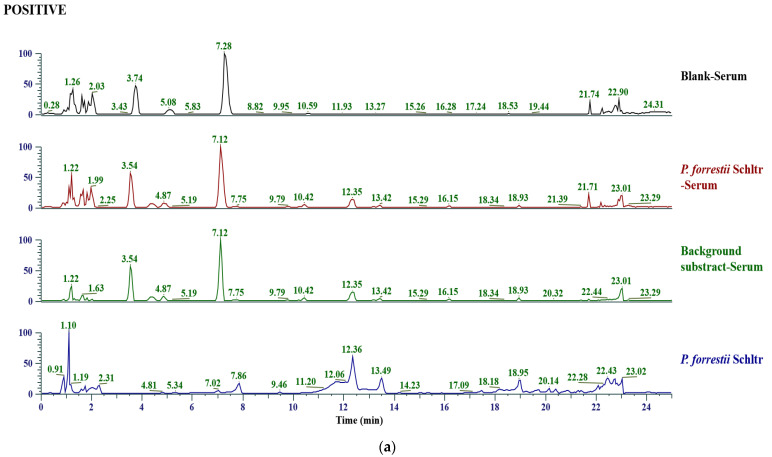

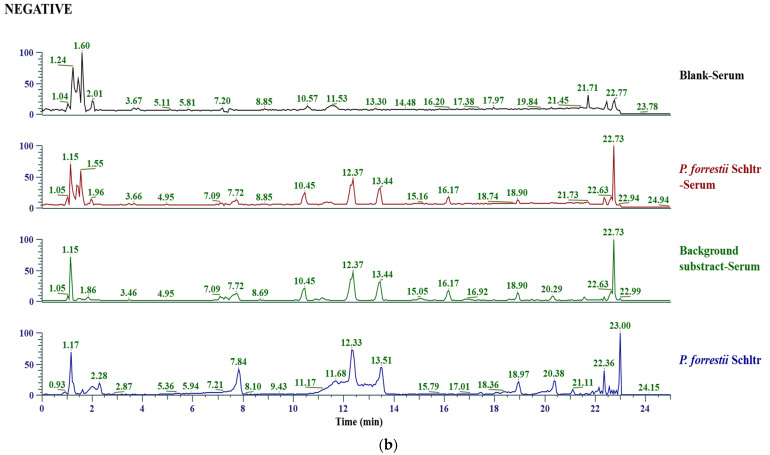
Total ion chromatograms of the *Periploca forrestii* Schltr. extracts and the plasma samples collected after drug administration in model rats: (**a**) positive ion mode; (**b**) negative ion mode.

Total ion chromatograms (TIC, Figure 2) of the extracts of *P. forrestii* and the plasma samples collected after drug administration in rats were obtained by UHPLC/Q Exactive Plus Orbitrap high-resolution mass spectrum (HRMS). Chemical composition identification was performed on Thermo Compound Discoverer 3.2; a total of 42 compounds were identified in *P. forrestii* extracts and were confirmed by alignment with the controls or standards database. These compounds involved a variety of types such as phenolic acid, coumarin, flavone, alkaloid, and others. Among them, protocatechuic acid, neochlorogenic acid, esculetin, and 17 other total components were absorbed into the blood. The compounds’ identification information is shown in Table S1.

**Figure 3:**
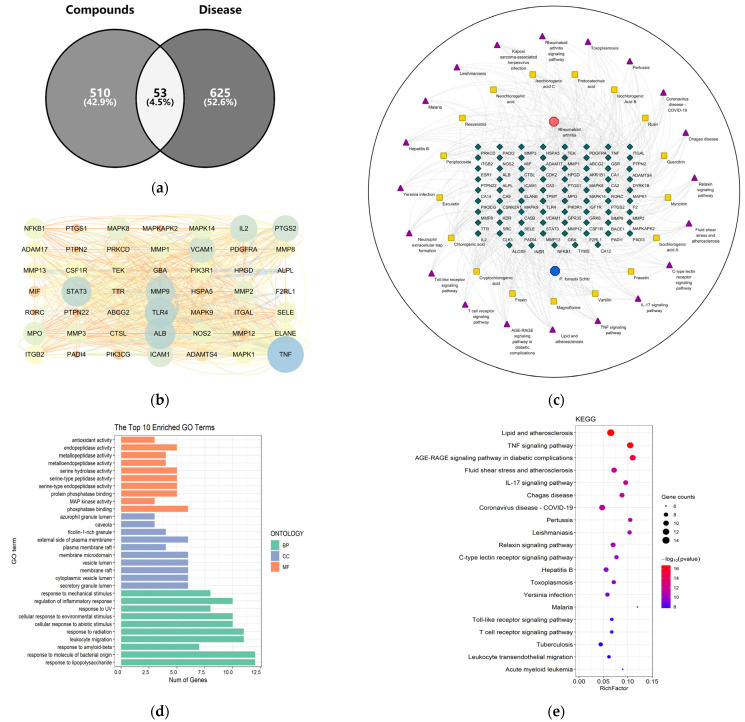
Network pharmacology analysis of *Periploca forrestii* Schltr. in the treatment of rheumatoid arthritis: (**a**) Venny diagram for screening common targets; (**b**) protein-protein interaction network diagram; (**c**) “*Periploca forrestii* Schltr.-Components-targets-pathways-Rheumatoid arthritis” network diagram; (**d**) GO enrichment analysis; (**e**) KEGG enrichment analysis.

There were a total of 574 targets for the 17 components absorbed into the blood by rats (after removing duplicates, Table S2), while there were 678 targets related to “RA” in the OMIM, Gene Cards, and TTD databases (after removing duplicates, Table S3). After standardization, a total of 53 targets were obtained from the intersection of the components and the disease (Figure 3a). Potential protein-protein interaction (PPI) relationships were analyzed using the String database, and network visualization was achieved using Cytoscape 3.7.0 software. The results indicated that 23 targets, including TNF, ALB, TLR4, STAT3, MMP9, IL2, VCAM1, PTGS2, and others, may be the core targets of *P. forrestii* in the treatment of RA (Figure 3b, Table S4). All 17 components were associated with intersecting targets and could be considered an effective component group for the *P. forrestii* treatment of RA (Table S5); the “Component-Target-Pathway-Disease” network diagram is shown in Figure 3c. Meanwhile, Gene Ontology (GO) and Kyoto Encyclopedia of Genes and Genomes (KEGG) enrichment analyses were conducted on the treatment of RA with *P. forrestii* using R 4.0.3 software. The results showed that the predicted target genes for treating RA with *P. forrestii* mainly involve the AGE-RAGE signaling pathway, the TNF signaling pathway, the IL-17 signaling pathway, the RA signaling pathway, and others (Figure 3d,e, Table S6).

### 2.3. Multi-Tech Metabolomics Analyses

#### 2.3.1. System Stability and Reproducibility Analyses of UPLC-Triple-TOF-MS/MS and GC-MS

Figures S1 and S2 show examples of typical TICs in UPLC-Triple-TOF-MS/MS and GC-MS analysis, respectively. To assess system stability and reproducibility, one quality control (QC) sample was injected per eight samples, and six QC samples were analyzed for the entire batch.

**Figure 4:**
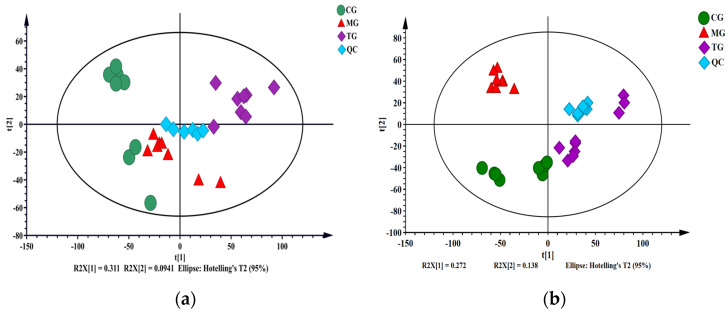
Principal component analysis distribution of all samples from (**a**) UPLC-Triple-TOF-MS/MS metabolomics and (**b**) GC-MS metabolomics. Note: CG means control group; MG means model group; TG means *Periploca forrestii* Schltr. extracts treatment group.

UPLC-Triple-TOF-MS/MS: By overlapping the TICs of the QC sample and comparing them (*n* = 6, Figure S3), it can be clearly seen that the intensity and retention time of each color spectrum peak of the QC sample basically overlap, and there is no significant deviation error. This indicates that the overall stability of the analysis system and the good state of the instrument are maintained throughout the entire sample analysis process [31,32]. Principal component analysis (PCA) was performed on all metabolites obtained to observe the overall distribution between each sample and the stability of the entire analysis process, while evaluating the system stability, as shown in Figure 4a. All QC samples are tightly clustered near the origin, indicating the good repeatability of this experiment [32,33]. In addition, the relative standard deviation (RSD) values of metabolites in all QC samples were calculated and the distribution of RSD was plotted. RSD reflects the degree of dispersion of a set of data and is typically used to measure the accuracy of analytical methods. As can be seen, 99% of the peak RSD is less than 30%, accounting for over 99% of the total peak area (Figure S4), indicating that the experimental operation and instrument conditions are relatively stable.

GC-MS: A mixed plasma sample was taken, and one QC sample was prepared according to the plasma sample processing method. Five consecutive injections were conducted for analysis. The number of chromatographic peaks in the TIC plot was obtained and compared after five injections, and ten main common peaks were examined. The results (Figure S5a) showed that the number of chromatographic peaks was consistent, and the RSD of the common peak area was between 2.1–10.6%, indicating that the instrument had good precision [33]. A plasma sample was taken, and five parallel samples were prepared according to the plasma sample processing method for injection analysis. The number of chromatographic peaks in the TICs were compared and the 10 main common peaks were examined. The results (Figure S5b) showed that the number of chromatographic peaks was consistent, and the RSD of the common peak area was between 1.8–11.4%, indicating the good repeatability of the method [33]. A plasma sample was taken and prepared according to the plasma sample processing method. The sample was injected six times within 24 h (0, 2, 6, 12, 18, 24 h). The number of chromatographic peaks in the TIC plot were compared and obtained after the six injections and the ten main common peaks were examined. The results (Figure S5c) showed that the number of chromatographic peaks was consistent, and the RSD of the common peak area was between 3.3–12.4%, indicating that the sample was stable after 24 h of derivatization [33]. One QC sample from each batch for analysis was randomly selected, the number of chromatographic peaks in the TIC plot obtained after each injection was compared and the RSD of the 10 main common peak areas were examined. The results (Figure S5d) showed that the number of chromatographic peaks was basically consistent, with RSDs ranging from 5.0% to 14.4%. In addition, PCA analysis was conducted on all samples to observe the overall distribution between each sample and the stability of the entire analysis process. All metabolites obtained were subjected to PCA analysis, and all QC samples were tightly clustered near the origin, indicating the good repeatability of this experiment (Figure 4b) [33,34].

Hence, the experiment had good repeatability, and the instrumental analysis system and the test data were stable and reliable [35].

#### 2.3.2. Multivariate Statistical Analysis

**Figure 5:**
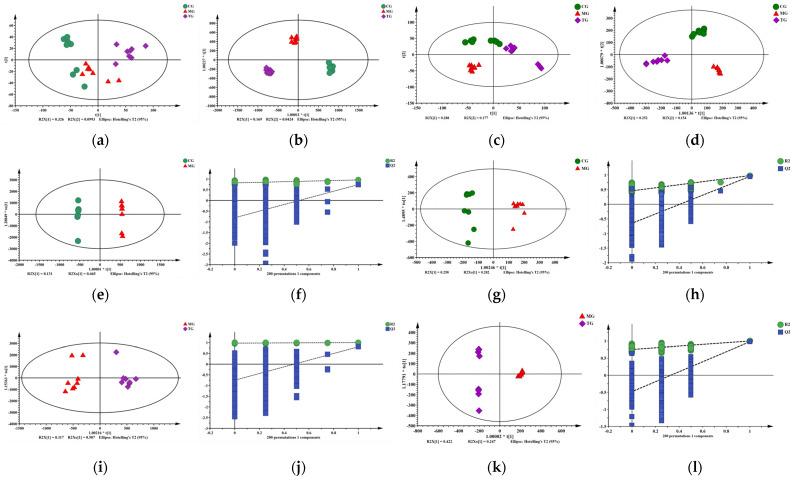
Multivariate statistical analysis. Note: CG-MG-TG: UPLC-Triple-TOF-MS/MS: (**a**) PCA, (**b**) OPLS-DA; GC-MS: (**c**) PCA, (**d**) OPLS-DA; CG-MG: UPLC-Triple-TOF-MS/MS: (**e**) OPLS-DA, (**f**) Permutation; GC-MS: (**g**) OPLS-DA, (**h**) Permutation; MG-TG: UPLC-Triple-TOF-MS/MS: (**i**) OPLS-DA, (**j**) Permutation; GC-MS: (**k**) OPLS-DA, (**l**) Permutation. Note: CG means control group; MG means model group; TG means *Periploca forrestii* Schltr. extracts treatment group.

A pattern recognition program was used to detect subtle metabolomic differences among different experimental groups and analyze the plasma UPLC-Triple-TOF-MS/MS and GC-MS data. PCA is an unsupervised data analysis method, which can be used to observe the overall distribution between samples and the stability of the entire analysis process. Therefore, PCA was first used to analyze the metabolic profiles of the CG, MG, and TG groups. The MG and TG groups were considerably separated. The results of this study indicated that the metabolites in the AA model rats were substantially altered after intervention with *P. forrestii* (Figure 5a,c). R^2^X determines the quality of the PCA model. Table S7 shows that R^2^X = 0.512, which is >0.5. Hence, the model is reliable.

The PCA showed the overall distribution trend among all groups. However, the endogenous metabolites accounting for the differences among groups are, as yet, unidentified [15]. Therefore, a supervised orthogonal partial least squares data analysis (OPLS-DA) was performed to classify samples and identify the metabolites that significantly contributed to the classification. OPLS-DA was performed to validate the separation of the metabolic profiles between the MG and CG groups and between the MG and TG groups. The score chart comparing MG and CG is shown in Figure 5e,g. The OPLS-DA model was subjected to 200-response permutation testing to determine whether it was overfitting. A permutation diagram (Figure 5f,h) was obtained; all Q^2^ (R^2^) on the left were lower than the original value on the right and the blue regression line where Q^2^ was located intersected the ordinate below zero. Therefore, the OPLS-DA model was reliable and there was no overfitting. The score chart comparing MG and TG is shown in Figure 5i,k. The permutation diagram (Figure 5j,l) showed that this model resembled the aforementioned one. Moreover, the TG group was distributed in the central region between the MG and CG groups (Figure 5b,d). As the modeling measurements continued during the experiment, the levels of small molecule metabolites dramatically changed in the MG group. After intervention with *P. forrestii*, the small molecule metabolite levels returned to normal.

#### 2.3.3. Differential Metabolite Analysis

The relative differences in marker metabolites were screened to explain the therapeutic mode of action of *P. forrestii*. The S-plot and volcano plots from the OPLS-DA analysis screened the differential metabolites. Those with variable important in projection (VIP) > 1 and *p* < 0.05 for the *t*-test were screened out as potential biomarkers for further identification; for example, graphical description with UPLC-Triple-TOF-MS/MS analysis results. In the S-plot load diagram (Figure S6a,b), the VIP magnitude and the impact on classification increased with distance from the origin. The volcano plot (Figure S6c,d) illustrated the *p*- and fold change (FC) values. The red origins represent significantly upregulated differential metabolites, the blue origins represent significantly downregulated differential metabolites, and the gray dots represent nonsignificant differential metabolites.

**Figure 6:**
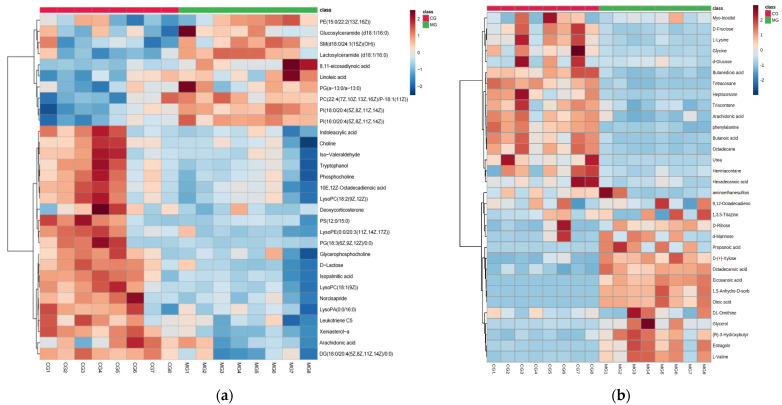
Heatmaps of the significantly differential metabolites. Metabolomics profile between the CG and MG groups showed a clear separation in the heatmap for UPLC-Triple-TOF-MS/MS (**a**) and GC-MS (**b**). Note: *n* = 8, per group; CG means control group; MG means model group. The abscissa indicates the sample name, and the ordinate indicates the differential metabolite. The color from green to red indicates the abundance of expression of metabolites from low to high, that is, the redder color indicates the higher expression abundance of differential metabolites.

Sixty-five potential plasma biomarkers were identified when the MG and CG groups were compared (Table S7). We also conducted hierarchical clustering of the expression levels of all significant metabolites. The heatmaps (Figure 6) showed that CG and MG are grouped into two clusters. This finding was consistent with the results of the PCA model, suggesting that the metabolites were reasonably screened, and revealed their metabolic variation. Twenty-seven endogenous metabolites were upregulated while thirty-eight endogenous metabolites were downregulated in the MG group. The putative biomarkers were mainly associated with glycerophospholipids, sphingolipids, saccharides fatty acyls, organonitrogen compounds, indoles, and their derivatives.

After intervention with the *P. forrestii* extracts, a total of 38 potential markers were restored to normal levels, including LysoPC(18:1(9Z)), chenodeoxycholic Acid, glycerophosphocholine, indoleacrylic acid, estragole, L-valine, butanoic acid, urea, glycerol, aminoethanesulfonic acid, glycine, and others (Table 1). Therefore, *P. forrestii* extracts may prevent pathological processes in RA rats by regulating disorders of the biomarkers of these putative metabolic pathways.

#### 2.3.4. Analysis of Metabolic Pathways

A biological pathway analysis was performed based on the KEGG. There are 23 main biochemical pathways related to RA, including phospholipid metabolism, fatty acid metabolism, amino acid metabolism, fatty acid metabolism, amino acid metabolism, glucose metabolism, and others. Table S8 shows the main metabolic pathway.

**Figure 7:**
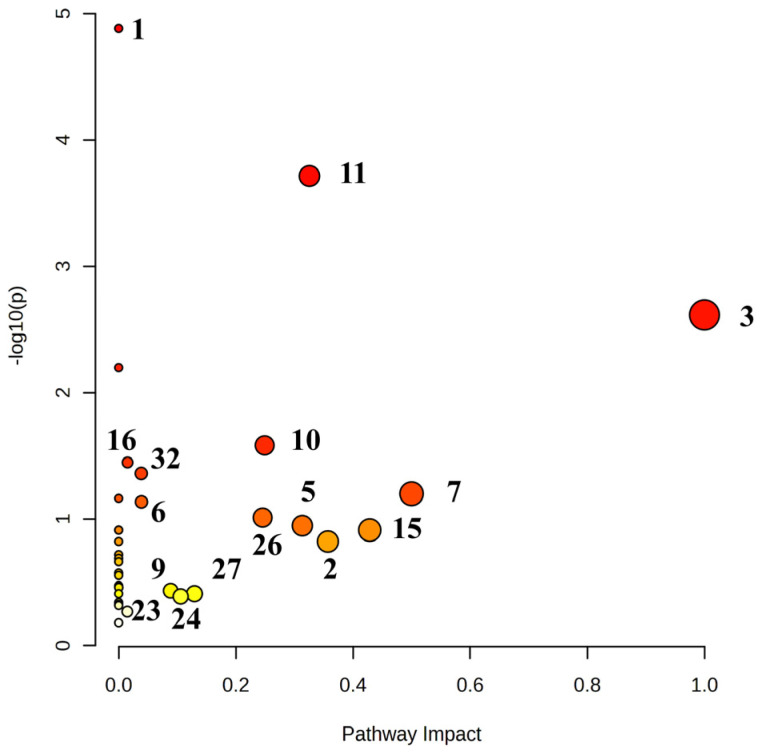
Total metabolic pathway related to *Periploca forrestii* Schltr. extracts restored according to MS analyses and MetaboAnalyst database. The pathways represented by the marked numbers in the figure correspond to Table 2 (Impact > 0). Note: The colors varying from yellow to red indicate an increasing significance [−log(*p*)] of metabolites in the data.

A total of 38 biomarkers found through UPLC-Triple-TOF-MS/MS and GC-MS metabolomics technologies were imported to the MetaboAnalyst 5.0 database for metabolic pathway analysis. After treatment with *P. forrestii* extracts, biosynthesis of unsaturated fatty acids, glycerophospholipid metabolism, linoleic acid metabolism, aminoacyl-tRNA biosynthesis, glycerolipid metabolism, sphingolipid metabolism, phenylalanine, tyrosine and tryptophan biosynthesis, and others were restored. The GC-MS technique was used to supplement the effect of *P. forrestii* extracts on the metabolites of AA rats. The results showed that *P. forrestii* extracts could regulate the metabolic pathways of Amino acid metabolism, glycerol phospholipid metabolism, fatty acid metabolism, glycolysis/gluconeogenesis, and others by jointly regulating amino acids, sugars, lipids, and other metabolites, thus playing a therapeutic role in RA. The analysis results are shown in Table 2 and Figure 7.

Identification of differential variables found in metabolomics comparison between AA rats and normal rats. The UPLC-Triple-TOF-MS/MS analysis method determined the changes in arachidonic acid metabolism, unsaturated fat acid biosynthesis, glycine, and serine, and threonine metabolism, glyceride metabolism, and primary bile acid biosynthesis were found using the GC-MS method. However, no changes in glucose metabolism (glycolysis/gluconeogenesis, pentose phosphate pathway, mutual conversion of pentose and glucuronate, galactose metabolism, synthesis, and degradation of ketones) using the GC-MS method were found.

After *P. forrestii* intervention treatment, some metabolites that underwent changes due to the disease underwent significant regression changes again. Overall, a total of 38 metabolites in the plasma of TG group rats underwent callback (Table 1, *p* < 0.05, compared with MG), mainly including lipids (glycerol phospholipids, arachidonic acids, fatty acids), amino acids, sugars, and others. Among them, sorted by VIP value, five out of the top ten metabolites affected (VIP ≥ 1.72) belonged to lipid metabolites (10E, 12Z Octadecadenoic acid, DG (18:0/20:4 (5Z, 8Z, 11Z, 14Z)/0:0), Iso Valeraldehyde, Arachidonic acid, and LysoPC (18:1 (9Z))), indicating that lipid metabolites play an important role in the metabolic regulation of the *P. forrestii* treatment of AA rats. The metabolic pathway results obtained after enrichment analysis also showed that four out of the top five metabolic pathways (−log(*p*) *>* 1.6) belonged to lipid metabolism (biosynthesis of unsaturated fatty acids, glycerophospholipid metabolism, linoleic acid metabolism, and glyceroolipid metabolism). Lipid metabolism is closely related to immune function, energy transmission, and inflammatory response in the body. Therefore, it can be speculated that *P. forrestii* may improve immune disorders and inhibit inflammatory response by adjusting lipid metabolism. *P. forrestii*, like other TCMs, plays an anti-RA metabolic regulatory role through multiple effects, with a greater emphasis on lipid metabolism. The difference in the mechanism of anti-RA action between *P. forrestii* and other types may be related to different substance compositions.

### 2.4. Pharmacodynamic Substances of the Periploca forrestii Schltr. Extracts against Rheumatoid Arthritis

**Figure 8:**
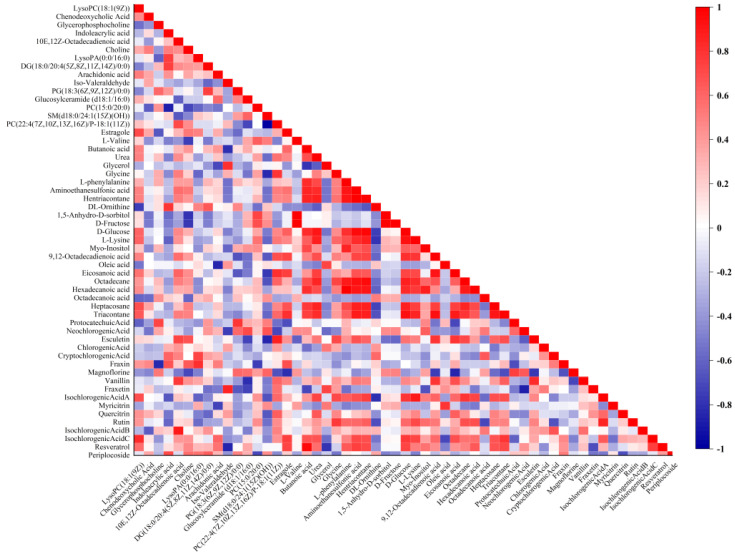
Correlation between exogenous blood-absorbed components and endogenous metabolites in model rats after administration of *Periploca forrestii* Schltr. extracts by Pearson correlation analysis.

The association between 17 exogenous blood-entry components and 38 endogenous metabolites was analyzed in AA rats after the administration of *P. forrestii* extracts to obtain pharmacodynamic substances of the *P. forrestii* extracts against RA. Correlation of the peak areas of the active ingredient groups of *P. forrestii* extracts for RA treatment with the peak areas of the different metabolites regressed after treatment using the Pearson correlation analysis. It is generally accepted that 0.6 ≤ |r|≤ 1 is a strong correlation, and an ingredient with six or more metabolite correlations is considered likely to be a pharmacophoric substance. A correlation plot of highly correlated components and metabolites is shown in Figure 8. There were five highly correlated components of *P. forrestii* extracts, including esculetin, magnoflorine, isochlorogenic acid A, rutin, isochlorogenic acid C, and resveratrol, and they could be considered a potential pharmacodynamic substance for the treatment of RA by the *P. forrestii* extracts.

## 3. Discussion

This study preliminarily reveals that the treatment of RA by *P. forrestii* extracts involves multiple components, multiple targets, multiple signaling pathways, and biological processes, and its main active components act on TNF, ALB, TLR4, STAT3, MMP9, IL2, VCAM1, PTGS2, and other proteins by regulating the AGE-RAGE signaling pathway, TNF signaling pathway, IL-17 signaling pathway, RA signaling pathway, and other related pathways. This may be one of the important mechanisms for reducing inflammatory response and treating RA [36].

In the previous in vitro and in vivo studies of the research group, the results of the in vitro MH7A cell anti-inflammatory experiment showed that [23] chlorogenic acid and 1,3-diffeoylquinic acid can significantly reduce COX-2 (i.e., PTGS2), NF-κB p65, and other protein levels. The results of the PK-PD research show that chlorogenic acid, cryptochlorogenic acid, and neochlorogenic acid can treat RA by inhibiting the secretion of IL-1β, RF, and TNF-α [24,37]. This is reciprocally corroborated with the prediction results from network pharmacology and also complemented by experimentation, combined with validation, further illustrating that caffeoylquinic acid components in *P. forrestii* extracts may be effective in the treatment of RA.

The results of this study show that, relative to untreated RA rats, *P. forrestii* extracts significantly reduced paw swelling and ankle joint circumference, repaired cartilage tissue, decreased inflammatory cell infiltration, and significantly lowered plasma RF, TNF-α, and IL-1β. Thus, the experiment herein successfully replicated the AA model and confirmed that *P. forrestii* has anti-RA efficacy. Figure 1 showed that the physiological status of the RA model rats did not fully return to normal levels after therapeutic drug intervention possibly because RA is a chronic degenerative disease and induces irreversible long-term damage. Animal pharmacodynamic studies rely on limited clinical biochemical and physical evaluation indicators. Hence, it is difficult to establish the overall efficacy of TCM. Metabolomics compares changes in the metabolite levels between the drug intervention and animal disease model groups, seeks biomarkers closely associated with the disease, evaluates overall TCM efficacy, and examines its modes of action [38].

Metabolomics can comprehensively study endogenous metabolites in complex organisms affected by pathology or after drug intervention, and integrate relevant metabolite information with metabolomics databases to provide changes in the metabolic network of the life system [39]. At present, metabonomics is widely used to clarify the pathogenesis of the COVID-19 virus [40], personalized medicine [41], biomarker discovery [42], and synthetic biology [43]. This research approach coincides with the overall thinking of TCM and can serve as an effective tool for discovering disease biomarkers and exploring the complex relationship between life systems and drugs [44]. Over the years, metabolomics has been widely applied in the study of TCM for the prevention and treatment of RA. For example, in the studies of *Astragalus membranaceus* var. *mongholicus* (Bunge) P.K.Hsiao (Huang-Qi) [45], *Atractylodes lancea* (Thunb.) DC (Cang-Zhu) [46], *Caulophyllum robustum* Maxim (Lei-Ye-Mu-Dan) [47], *Bassecoia hookeri* (C. B. Clarke) V. Mayer and Ehrend. (Yi-Shou-Cao) [48], and others, the UPLC-Q TOF-MS method has been used to analyze the metabolomics of serum, plasma, and urine, and to elucidate the metabolic regulatory mechanisms of these plant drugs for the prevention and treatment of RA from a metabolic perspective.

After intervention with Huang-Qi extract in AA rats, thirteen and twenty-one metabolites in urine and serum were regulated, mainly involving five metabolic pathways such as tryptophan metabolism and fatty acid metabolism. This indicates that Huang-Qi extract has the effect of inhibiting inflammation and improving oxidative stress status [45]. After intervention with Cang-Zhu extract, the levels of taurocholic acid, taurocholic acid, and 11-deoxycortisol in AA rats decreased. It is speculated that Cang-Zhu may improve the immune function of the body by affecting the biosynthesis of primary bile acids and steroid hormones [46]. Lei-Ye-Mu-Dan can restore the urine metabolism markers and thirteen plasma metabolism markers of collagen-induced arthritis rats to normal levels, which mainly involve pathways such as lipid metabolism, energy metabolism, and amino acid metabolism [47]. Yi-Shou-Cao extract can restore 19 biomarkers such as valine, arachidonic acid, and proline to the control group level, indicating that Yi-Shou-Cao extract can improve immune disorders and inhibit inflammatory reactions in the body [48].

In this study, we used UPLC-Triple-TOF-MS/MS and GC-MS plasma metabolomics to identify 38 potential differentially expressed biomarkers between the MG and CG groups. The enrichment analysis (Table 2) showed that these putative biomarkers are mainly involved in the metabolism of glycerophospholipid, linoleic acid, tryptophan, and others. After identifying the difference variables found in the comparison between the metabolic groups of AA rats and normal rats, the LC-MS analysis method and the GC-MS method found the changes in arachidonic acid metabolism, unsaturated fat acid biosynthesis, glycine, serine and threonine metabolism, glycerol lipid metabolism, and primary bile acid biosynthesis. However, LC-MS did not find changes in glucose metabolism (glycolysis/gluconeogenesis, pentose phosphate pathway, mutual conversion of pentose and glucuronate, galactose metabolism, synthesis, and degradation of ketones). This result indicated that the metabolomics method of LC-MS analysis can be mutually validated with the metabolomics method based on GC-MS analysis, while also complementing each other in searching for disease-related metabolic changes.

The liver is the main source of plasma glycerophospholipids. These biomolecules are important constituents of liver cells and mitochondrial membranes and participate in the pathogenesis of tumors and inflammatory diseases. Certain metabolomics studies have disclosed that abnormal glycerophospholipid metabolism in AA rats is associated with immune responses. LPC and LPE are major signaling molecules that regulate the inflammatory response by inhibiting proinflammatory secretions [24]. Our results showed that the glycerophospholipid metabolites including LysoPE(0:0/20:3(11Z,14Z,17Z)), LysoPC(18:1(9Z)), LysoPC(18:2(9Z,12Z)), PE(20:2(11Z,14Z)/22:4(7Z,10Z,13Z,16Z)), PC(15:0/20:0), PC(22:4(7Z,10Z,13Z,16Z)/P-18:1(11Z)), PE(15:0/22:2(13Z,16Z)), and others are differentially expressed in AA. Thus, the phospholipid metabolism pathways were markedly perturbed. Previous studies on autoimmune hepatitis have shown that lysoPE was significantly downregulated in the plasma of concanavalin A-treated mice [49]. The LPC levels were significantly reduced in human patients and AA mouse models [50]. Our research showed that most LPC biomarkers were reduced in the AA model group. This finding was consistent with earlier reports.

Sphingolipid metabolism regulates numerous cellular behaviors related to health and disease. Surowiec used LC-MS metabolomics and discovered that the sphingomyelin (SM) content was relatively elevated in patients with early RA [51]. Furthermore, their SM levels continued to rise with RA progression. Thus, it was suggested that SM is a powerful RA predictor. In this study, (SM(d18:0/24:1(15Z)(OH))) was also significantly increased in the untreated RA rats relative to the treated groups. Ceramide is a sphingomyelin decomposition product. A previous study [52] stated that elevated ceramide directly affects mitochondria, causes abnormal macrophage function, and promotes inflammation. The results of this study showed that plasma glucosylceramide (d18:1/16:0) and lactosylceramide (d18:1/16:0) were high in RA model rats. Lin found that ceramide was essential for epidermal homeostasis and played vital roles in hair follicle structure and function [53]. Abnormal ceramide levels can cause alopecia. Therefore, disturbances in sphingolipid metabolism may cause hair loss in AA rats. This phenomenon was observed in the AA model rats of the present study.

Fatty acids are closely associated with inflammation. Free fatty acids activate macrophages, release numerous inflammatory factors, and participate in the onset and progress of inflammation [54]. Palmitic and linoleic acids are the main free fatty acids in plasma. Here, the plasma isopalmitic acid levels were relatively low, and the plasma linoleic acid content was comparatively high in the RA rats. Therefore, the free fatty acid metabolism disorders were related to RA. The linoleic acid oxidation products 10E,12Z-octadecadienoic acid and 8,11-eicosadiynoic acid were also detected in the plasma of RA rats. Linoleic acid is a substrate for lipoxygenase, cyclooxygenase, P450 enzyme, and non-enzymatic reactions. It has been reported in the literature that linoleic acid oxidation products enhance the expression of tumor necrosis factor alpha (TNF-α) in Kupffer cells and promotes inflammatory responses [55]. Linoleic acid is an essential fatty acid for humans. It is a precursor of arachidonic acid via the action of Δ6-dehydrogenase and Δ5-dehydrogenase. Arachidonic acid is a polyunsaturated essential fatty acid known to induce inflammation. The reaction of arachidonic acid with methyl cyclooxygenase produces prostacyclin, TXA2, leukotriene, and other compounds. This mechanism is an immune response to inflammation. Arachidonic acid serves as a signaling molecule [56]. In the RA model in this study, the linoleic acid and leukotriene C5 levels were relatively reduced, and the arachidonic acid content was comparatively increased, possibly because of certain changes in enzymatic activity induced by the inflammatory response. Pang discovered, via plasma metabolomics, that the incidence of RA was correlated with arachidonic and linoleic acid metabolism [57]. The results of the present study were consistent with those reported in the literature.

The application of multi omics technology in multiple fields of life sciences is becoming increasingly widespread. For example, in the study of prostate cancer, by integrating transcriptome and metabolome data, the main metabolic pathways and potential biomarkers that cause prostate cancer hyperplasia are revealed [58]. In research pertaining to the COVID-19 mechanism, the COVID-19 SARS-CoV-2 database, COVID-19 patients’ genome data, transcriptome data, microbiome data, drug treatment, and other data information have been integrated to explore the pathogenesis of COVID-19 [59]. By integrating transcriptome, metagenomic, proteomic, and metabolomic data, aging mechanism research has been conducted to evaluate biological age and screen biomarkers related to aging [60]. Genomics is used to study the composition, overall structure, and role in the genetic processes of DNA. Transcriptomics is mainly used to study the expression patterns of genes. Proteomics is used to study the structure and function of proteins, as well as their interactions with other proteins. Metabolomics is used to study the production and consumption of metabolites in organisms, as well as their interactions. On the basis of this study, further multi omics methods can be combined in the future to elucidate the anti-RA mechanism of *P. forrestii* from multiple perspectives.

This study used LC-MS and GC-MS for metabolomics studies and obtained metabolic markers for *P. forrestii*’s anti-RA effect. This information can provide a basis for the dosage selection for *P. forrestii* clinical application in terms of metabolic mode of action and provides a foundation for further clinical application. This study is the first to report on the plasma metabolomics of *P. forrestii* against RA, filling the gap in the metabolic regulation mechanism of *P. forrestii.* In terms of detection methods, compared with the research method of a single detector, the results of LC-MS and GC-MS complement each other, and the results of metabolite detection are more comprehensive. Especially for compounds related to sugar metabolism, they can be detected on GC-MS, but cannot be detected on LC-MS. The correlation analysis between endogenous metabolites and exogenous blood-absorbed components screened out the effective substances of *P. forrestii* against RA. It can provide an experimental basis for further pharmacodynamic research, molecular mechanism verification, and even the development of new dosage forms in the future. However, this study only explained the anti-RA action mechanism of *P. forrestii* from the perspective of metabolomics, and the exploration stayed at the metabolic phenotype stage. When it exerts its anti-RA effect, its ability to affect the protein level or even the gene level is not yet clear. Moreover, this study only used plasma for detection, and the study of metabolic mechanisms is not comprehensive. In the future, based on this study, it can be further combined with other high-throughput technologies (e.g., proteomics, transcriptomics, 16sRNA, and others) to provide a more comprehensive explanation for the mechanism of *P. forrestii*’s anti-RA action. As a systemic immune disease, RA affects many biological processes and affects a variety of organs. In addition to blood, RA could also affect urine, feces, joint synovial fluid, and even the heart. Further multi-sample metabolomic research can be carried out to provide a more comprehensive explanation of the metabolic regulatory mechanism. At the same time, we can also combine targeted omics methods or molecular biology methods to verify its mechanism of action and elucidate its anti-RA effect at the molecular level. In the future, after the molecular mechanism is clarified, it may provide a basis for clinical precision medicine and personalized treatment of *P. forrestii*.

## 4. Materials and Methods

### 4.1. Herbal and Chemicals

*P. forrestii* was purchased from Wandongqiao market in Guiyang in Guizhou Province of China and identified as *Periploca forrestii* Schltr. in the *Periploca* genera of the *Asclepiadaceae* families by Associate Prof. Chunhua Liu of the Department of Pharmacognosy of Guizhou Medical University, Guiyang, China. Rat IL-1β, RF, and TNF-α ELISA kits were purchased from Nanjing Jiancheng Biotech Company, Nanjing, China. Freund’s complete adjuvant, N-methyl-N(trimethyl silyl)trifluoroacetamide (MSTFA), and trimethylchlorosilane (TMCS) were purchased from Sigma-Aldrich, St. Louis, MO, USA. *L*-2-chlorophenylalanine was purchased from Shanghai Hengchuangsheng Technology Co., Ltd., Shanghai, China. LysoPC17:0 was purchased from Avanti Polar Lipids, Inc., Alabaster, AL, USA. Methanol, formic acid, water, and acetonitrile were purchased from CNW Technologies GmbH, Duesseldorf, Germany. Pyridine (No water level, purity ≥ 99.9%) and methoxamine hydrochloride were purchased from Shanghai Aladdin Biochemical Technology Co., Ltd., China. Other reagents were of analytical purity level.

### 4.2. Periploca forrestii Schltr. Extraction

Six kilograms of *P. forrestii* was extracted thrice with 8× the amount of 70% (*v*/*v*) ethanol. The extraction times were 1.5, 1.0, and 1.0 h. The extracts were then filtered, and the filtrates were combined and concentrated to 1 g/mL under reduced pressure. The final concentration was calculated according to the amount of raw material. The mixture was extracted thrice with saturated *n*-butanol. The *n*-butanol extracts were combined, and the *n*-butanol was recovered under reduced pressure. The residue was dried under a vacuum at 45 °C. The final extraction rate was 7.68% [61].

### 4.3. Model Construction and Drug Administration

Forty male Sprague-Dawley rats with a mean initial body weight of 200 ± 20 g were purchased from the Experimental Animal Center of Guizhou Medical University (Certificate No. SCXK (Guizhou) 2018-0001). The animals were housed in air-conditioned animal quarters at a controlled temperature (20–25 °C) and relative humidity (40–70%). The animal studies were approved by the Experimental Animal Centre of Guizhou Medical University and conducted in strict accordance with the guidelines of the National Institutes of Health for the Care and Use of Animals in China. The rats were randomly divided into 4 groups of 8 rats each. These were the control (CG), the model (MG), the treatment (TG, *P. forrestii* treatment), and the positive (PG, niportgium glycosides treatment) groups. All rats were injected with 0.1 mL (10 mg/mL) FCA in the right posterior pad to induce inflammation. The normal group was injected with an equal volume of normal saline. On day 7 after immunization, the rats were re-immunized. On day 21 after modeling, the TG group was intragastrically administered *P. forrestii* extracts at 87 g/kg twice daily for 14 d. The PG group was intragastrically administered niportgium glycosides at 102 mg/kg twice daily for 14 d. The rats in the CG and MG groups were fed equal volumes of 0.5% (*w*/*v*) sodium carboxymethylcellulose solution. The components of the *Tripterygium wilfordii* polyglycoside tablets and the *P. forrestii* extracts were prepared with 0.5% (*w*/*v*) sodium carboxymethylcellulose solution.

### 4.4. Basic Physiological Parameters

Paw swelling and ankle circumference were selected to evaluate arthritis model development and *P. forrestii* treatment efficacy. Paw swelling was measured with a PV-200 Paw Swell Tester (Chengdu Taimeng Biological Co., Ltd., Chengdu, China). On days 0, 1, 3, 5, 7, 9, 11, 17, 19, 21, 24, 26, 28, 30, 32, and 34 after modeling, the circumferences of the ankle joints were measured with a soft ruler.

### 4.5. Quantification of Serum TNF-α and IL-1β and Histopathological Analysis

On day 35 after primary immunization, blood was collected to obtain plasma for the measurement of the inflammatory factors RF, TNF-α, and IL-1β with ELISA kits (Nanjing Jiancheng Biotechnology Co., Ltd., Nanjing, China). After the plasma samples were prepared, the rats were sacrificed. The right hind paws and ankle joints were excised and immersed in a formaldehyde solution. The tissues were then decalcified with EDTA-2Na, dehydrated with ethanol, embedded in paraffin, and stained with HE for pathological examination [62].

### 4.6. In Vitro and In Vivo Chemical Composition Identification

#### 4.6.1. In Vitro

One microgram of *P. forrestii* extracts was added to a conical flask with 25 mL of 50% methanol, sonicated for 30 min (40 Khz, 25 °C), and centrifuged at 15,000× *g* rpm for 10 min, and the supernatant was injected into the UHPLC-HRMS system with an injection volume of 2 μL.

#### 4.6.2. In Vivo

The sera of 8 animals in each group were mixed to obtain mixed drug-containing serum and blank serum to eliminate individual differences. An amount of 300 µL of 2% formic acid aqueous solution and 2.4 mL of methanol solution were added into 600 µL of mixed drug-containing serum and mixed blank serum. They were vortexed for 2 min, sonicated for 10 min, and centrifuged at 12,000× *g* rpm at 4 °C for 10 min to obtain the supernatant. The supernatant was blown dry at 37 °C on a nitrogen blower. The residue was added to 200 µL of a 50% methanol solution, vortexed for 2 min, sonicated for 10 min, centrifuged at 12,000× *g* rpm at 4 °C for 10 min, and the supernatant was taken into the instrument for analysis, with an injection volume of 2 µL.

#### 4.6.3. Apparatus Condition

The liquid phase system adopted Thermo Vanquish Horizon ultra-high performance liquid chromatography with a Thermo Hypersil Gold (150 mm × 2.1 mm, 1.9 μm) chromatographic column and ID filter (2.1 mm, 0.2 μm). The column temperature was 40 °C and the injector temperature was 4 °C. The mobile phases were gradient eluted with 0.1% formic acid water (A) −0.1% formic acid acetonitrile (B) at a flow rate of 0.35 mL/min, and the chromatographic elution procedure was as follows: 0–2 min, 5% B; 2–17 min, 5–25% B; 17–19 min, 25–30% B; 19–21 min, 20–95% B; 21–23 min, 95% B; 23–23.5 min, 95–5% B; 23.5–25 min, 5% B. The mass spectrometry system adopted Thermo Q Exactive Orbitrap Plus MS, with detection modes of ESI^+^&ESI^−^, and the sheath gas and AUX gas were N_2_. The mass spectrometry data collection software was Thermo Xcalibur 4.1. Other mass spectrometry parameters were as follows: spray voltage: 3.5 kV (+)/3.0 kV (−); capillary temp 300 °C; vaporiser temp: 350 °C; scan range: 70–1050; sheath gas: 40 arb; general method: Fullms-ddms2; AUX gas: 10 arb; resolution (MS1): 70,000; MS/MS resolution: 17,500; stepped NCE: 20, 40, 60.

### 4.7. Network Pharmacology

In the Swiss Target Prediction database (https://www.swisstargetprediction.ch/, accessed on 15 July 2023), compound action targets were predicted based on the structure of the absorbed-blood components, while Online Mendelian Inheritance in Man (OMIM, https://www.omim.org/, accessed on 15 July 2023), GeneCards (https://www.genecards.org/, accessed on 15 July 2023), and the Therapeutic Target Database (TTD, https://db.idrblab.net/ttd/, accessed on 15 July 2023) were used to obtain the relevant targets for RA. The normalization of target names was achieved by retrieving the gene symbol for each target based on the UniProt database (https://www.uniprot.org/, accessed on 15 July 2023). The intersection of constituent targets and disease targets was taken, and the intersection was determined by Venn diagram analysis using Venn diagrams online tool (https://bioinfogp.cnb.csic.es/tools/venny/, accessed on 15 July 2023). The potential protein-protein interaction (PPI) relationship was analyzed using the String database (https://cn.string-db.org/, accessed on 15 July 2023), the network was visualized, and the active ingredient groups were obtained based on Cytoscape 3.7.0. R 4.0.3 was used to conduct GO and KEGG enrichment analysis on the treatment of RA by *P. forrestii*.

### 4.8. Multi-Technical Metabolomics Analyses

#### 4.8.1. UPLC-Triple-TOF-MS/MS Metabolomics Analysis

The samples stored at −80 °C were removed and thawed. Then, 100 μL of plasma and 10 μL of each internal standard (*L*-2-chlorophenylalanine, 0.3 mg/mL; Lyso PC17:0, 0.01 mg/mL; both in methanol) were quantitatively transferred to clean centrifuge tubes, vortexed for 10 s, combined with 300 μL methanol-acetonitrile (2:1, *v*/*v*) protein precipitant, ultrasonically extracted in an ice water bath for 10 min, left to stand at −20 °C for 30 min, and centrifuged for 10 min (15,521× *g*; 4 °C). The supernatant was then filtered through a 0.22-μm organic-phase pinhole filter and analyzed by UPLC-Triple-TOF-MS/MS. QC plasma samples were prepared by mixing equal volumes of the extracts of all samples and processing them according to the aforementioned protocol. The volume of each QC sample was the same as those of the metabolites.

Chromatographic analysis was performed in a Waters Acquity UPLC system coupled with an AB SCIEX Triple TOF 5600 System (AB SCIEX, Framingham, MA, USA). Chromatographic separation was conducted at 45 °C on an ACQUITY UPLC BEH C_18_ column (100 mm × 2.1 mm, 1.7 μm, Waters Corp., Milford, MA, USA). The injection volume was 5 μL and the flow rate was 0.4 mL/min. The mobile phase consisted of solvent A (0.1% formic acid-water, *v*/*v*) and solvent B (acetonitrile-methanol, 2:3, *v*/*v*). An elution gradient was used (0–2 min, 5–20% B; 2–4 min, 20–25% B; 4–9 min, 25–60% B; 9–17 min, 60–100% B; 17–19 min, 100% B; 19–19.1 min, 100–5% B; 19.1–20.1 min, 5% B at a flow rate of 0.4 mL/min). For the MS analysis, sample mass spectrometry signals were collected in both positive and negative ion scanning modes using an electrospray ionization source (ESI). *L*-2-chlorophenylalanine and LysoPC17 were used to calibrate the mass spectrometer and as the reference mass, respectively. The mass spectrometry conditions were as follows: nebulizer gas, 40 PSI; auxiliary gas, 40 PSI; curtain gas, 35 PSI; source temperature, 550 °C (+) and 550 °C (−); ion spray voltage, 5500 V (+) and 4500 V (−); declustering potential, 100 V (+) and −100 V (−); collision energy, 10 eV (+) and −10 eV (−); interface heater temperature, 550 °C (+) and 600 °C (−). The *m/z* range was 70–1000. The collision energy was set to 30 eV for selected compound identification.

#### 4.8.2. GC-MS Metabolomics Analysis

One-hundred microliter of the thawed plasma sample was taken in a 1.5 mL EP tube; 250 μL of methanol was added. The mixture was vortexed for 3 min, ice-bathed for 10 min, and centrifuged at 10,000× *g* rpm for 10 min under 4 °C to obtain plasma methanol extracts. An amount of 250 μL of the plasma methanol extract was put into a GC sample bottle and dried with N_2_. Then, 50 μL of 15 mg/mL methoxamine pyridine solution was added, then mixed well, oximated for 1 h (70 °C). Subsequently 50 μL derivatization reagent (MSTFA:TMCS = 100:1, *v*/*v*) was put added, mixed well, and derivatized for 1 h (70 °C). After cooling, it was transferred to a 1.5 mL EP tube and centrifuged (15,000× *g* rpm, 10 min, 4 °C) to obtain the supernatant for GC-MS analysis. The preparation of QC samples is the same as the LC-MS metabolomics analysis.

Agilent 7890A/5975C-GC/MSD was used for GC-MS metabolomics with column HP-5MS (30 m × 0.25 mm, 0.25 μm) and the injection volume was 1 μL. The shunt mode was non-shunt, and the inlet temperature, Electron Ionization (EI) ion source temperature, and transmission line temperature were 270 °C, 230 °C, and 280 °C, respectively. The electronic voltage was 70 eV; the mass spectrometric scanning range was *m*/*z* 40–600; the program heating conditions started at 80 °C, and were maintained for 5 min; heated up to 260 °C at 6 °C/min and maintained for 3 min; heated up to 310 °C at 10 °C/min and kept for 5 min.

### 4.9. Anti-RA Pharmacodynamic Substances Screening of P. forrestii Extracts

The potential efficacy of the *P. forrestii* extracts and its endogenous metabolites in the treatment of the partial effects of the RA process can be obtained from the above experiments. This section further develops Pearson’s correlation analysis to discuss the association between exogenous components and endogenous metabolites in AA rats after *P. forrestii* extract was given, with the expectation of screening out efficacy substances that are closely related to disease treatment.

### 4.10. Data Processing and Statistical Analysis

The original UPLC-Triple-TOF-MS/MS and GC-MS data were baseline-filtered, peak-identified, retention time-corrected, peak-aligned, and normalized by Progenesis QI v. 2.3 (Nonlinear Dynamics, Newcastle, UK) or XCMS Online (https://xcmsonline.scripps.edu, accessed on 1 May 2021). A data matrix containing the retention time, the mass-to-charge ratio, and the peak intensity was obtained. The normalized data matrix was imported into SIMCA v. 14.0 (Umetrics, Umea, Sweden) for multivariate statistical analysis. The latter first used unsupervised PCA to observe the sample distribution population and the stability of the entire analytical process. A supervised partial least squares data analysis (PLS-DA) and an OPLS-DA were conducted to distinguish the overall differences among groups in terms of their metabolic profiles. The importance of the variables in the projection (VIP) was ranked in the OPLS-DA analysis according to the overall contribution of each variable to the model. The output was combined with that of Student’s *t*-test. VIP > 1 and *p* < 0.05 indicate important differentially expressed metabolites. The different variables were retrieved through databases such as Metlin, HMDB, and LipidMaps, and combined with secondary fragment ions for structural identification. According to the identifications, a metabolic pathway analysis was conducted in KEGG. Pathways with *p* < 0.05 were filtered as major metabolic pathways. One-dimensional statistical analyses include multiples of variation FC analysis, *t*-tests, and volcano plots.

## 5. Conclusions

In this study, we combined pharmacodynamics and plasma nontarget metabolomics to elucidate the therapeutic efficacy and modes of action of *P. forrestii* on AA rats. Through component analysis in vivo and LC/GC-MS metabolomics studies, it was found that *P. forrestii* prevented and treated RA mainly by affecting lipid metabolism processes. To the best of our knowledge, this is the first plasma metabolomics study on the therapeutic mechanism of *P. forrestii* extracts against RA in rats, and this information provides a basis for the selection of metabolic action modes for *P. forrestii* clinical application dosage. At the same time, potential pharmacological substances of *P. forrestii* that may exert anti-RA effects were discovered. The metabolic markers and pharmacological substances identified in this study for the treatment of RA with *P. forrestii* can provide an experimental basis for further research on pharmacoequivalence, molecular mechanism validation, and even the development of new dosage forms in the future. However, the limitation of this study is that it only narrates from the perspective of metabolic phenotype. In the future, multiple-omics studies can be conducted on this basis to further explore the anti-RA characteristics of *P. forrestii* at the gene and protein levels.

## Figures and Tables

**Table 1:** Potential plasma biomarkers identified in the *Periploca forrestii* Schltr. extracts treatment group compared to the model group and their relative levels (↑, upregulation; ↓, downregulation).

No	RT	Metabolites	VIP	Tend	
	(min)			MG ^a^	TG ^b^
LC 1	1.87	Indoleacrylic acid	1.22	↓	↑
LC 2	5.52	Chenodeoxycholic Acid	1.10	↓	↑
LC 3	6.14	Arachidonic acid	2.27	↓	↑
LC 4	6.17	10E,12Z-Octadecadienoic acid	4.52	↑	↓
LC 5	6.19	Glycerophosphocholine	1.27	↓	↑
LC 6	6.19	Iso-Valeraldehyde	2.52	↓	↑
LC 7	6.62	LysoPA(0:0/16:0)	1.07	↓	↑
LC 8	6.87	LysoPC(18:1(9Z))	1.82	↓	↑
LC 9	6.92	Choline	1.43	↓	↑
LC 10	8.09	PG(18:3(6Z,9Z,12Z)/0:0)	1.09	↓	↑
LC 11	12.60	Glucosylceramide (d18:1/16:0)	1.50	↑	↓
LC 12	13.99	PC(22:4(7Z,10Z,13Z,16Z)/P-18:1(11Z))	1.09	↑	↓
LC 13	14.01	PC(15:0/20:0)	1.01	↑	↓
LC 14	14.03	SM(d18:0/24:1(15Z)(OH))	1.33	↑	↓
LC 15	14.06	DG(18:0/20:4(5Z,8Z,11Z,14Z)/0:0)	2.63	↓	↑
GC 1	10.74	Estragole	1.42	↑	↓
GC 2	10.85	L-Valine	1.30	↑	↓
GC 3	11.57	Butanoic acid	1.30	↓	↑
GC 4	12.22	Urea	2.18	↓	↑
GC 5	12.36	Glycerol	1.92	↑	↓
GC 6	12.56	Aminoethanesulfonic acid	1.22	↓	↑
GC 7	13.10	Glycine	1.56	↓	↑
GC 8	20.08	L-phenylalanine	1.72	↓	↑
GC 9	21.48	Hentriacontane	1.25	↓	↑
GC 10	23.47	DL-Ornithine	1.28	↑	↓
GC 11	24.04	1,5-Anhydro-D-sorbitol	1.79	↑	↓
GC 12	24.36	D-Fructose	1.02	↓	↑
GC 13	25.20	D-Glucose	1.07	↓	↑
GC 14	25.28	L-Lysine	1.08	↓	↑
GC 15	27.86	Myo-Inositol	1.05	↓	↑
GC 16	29.15	Heptacosane	2.77	↓	↑
GC 17	29.90	9,12-Octadecadienoic acid	1.27	↑	↓
GC 18	29.91	Oleic acid	1.48	↑	↓
GC 19	33.06	Eicosanoic acid	1.06	↑	↓
GC 20	34.91	Hexadecanoic acid	1.06	↓	↑
GC 21	37.79	Octadecanoic acid	1.00	↑	↓
GC 22	38.22	Octadecane	1.50	↓	↑
GC 23	40.94	Triacontane	1.30	↓	↑

Note: MG ^a^: The change trend compared between the control group and the model group, ^a^ means there was a significant difference in the peak area between the two groups for this metabolite (*p* < 0.05); TG ^b^: The change trend compared between the model group and the *Periploca forrestii* Schltr. extracts treatment group, ^b^ means there was a significant difference in peak area between the two groups for this metabolite (*p* < 0.05).

**Table 2:** Main metabolic pathways involved in *Periploca forrestii* Schltr. extracts restored according to UPLC-Triple-TOF-MS/MS and GC-MS analyses and MetaboAnalyst database.

No	Pathway Name	Match Status	*p*	−log(*p*)	Holm *p*	FDR	Impact
1	Biosynthesis of unsaturated fatty acids ^a,b^	6/36	1.02 × 10^−5^	4.9934	0.0009	0.0009	0
2	Glycerophospholipid metabolism ^a^	5/36	1.57 × 10^−4^	3.8041	0.0130	0.0066	0.3257
3	Linoleic acid metabolism ^a,b^	2/5	2.23 × 10^−3^	2.6508	0.1832	0.0626	1.0000
4	Aminoacyl-tRNA biosynthesis ^b^	4/48	5.46 × 10^−3^	2.2631	0.4420	0.1146	0
5	Glycerolipid metabolism ^a,b^	2/16	2.42 × 10^−2^	1.6169	1.0000	0.4059	0.2492
6	Sphingolipid metabolism ^a^	2/21	4.03 × 10^−2^	1.3943	1.0000	0.5648	0.0385
7	Phenylalanine, tyrosine and tryptophan biosynthesis ^b^	1/4	6.06 × 10^−2^	1.2177	1.0000	0.6348	0.5000
8	Galactose metabolism ^b^	2/27	6.37 × 10^−2^	1.1956	1.0000	0.6348	0
9	Phosphatidylinositol signaling system ^a,b^	2/28	6.80 × 10^−2^	1.1674	1.0000	0.6348	0.0389
10	Glycine, serine and threonine metabolism ^a,b^	2/33	9.07 × 10^−2^	1.0425	1.0000	0.7600	0.2458
11	Arachidonic acid metabolism ^a,b^	2/36	1.05 × 10^−1^	0.9779	1.0000	0.7600	0.3135
12	Ascorbate and aldarate metabolism ^b^	1/8	1.18 × 10^−1^	0.9295	1.0000	0.7600	0
13	Valine, leucine and isoleucine biosynthesis ^b^	1/8	1.18 × 10^−1^	0.9295	1.0000	0.7600	0
14	Biotin metabolism ^b^	1/10	1.45 × 10^−1^	0.8390	1.0000	0.8113	0
15	Phenylalanine metabolism ^b^	1/10	1.45 × 10^−1^	0.8390	1.0000	0.8113	0.3571
16	Primary bile acid biosynthesis ^a,b^	2/46	1.58 × 10^−1^	0.8024	1.0000	0.8276	0.0076
17	alpha-Linolenic acid metabolism ^a^	1/13	1.84 × 10^−1^	0.7346	1.0000	0.9105	0
18	Arginine biosynthesis ^b^	1/14	1.97 × 10^−1^	0.7055	1.0000	0.9193	0
19	Butanoate metabolism ^b^	1/15	2.10 × 10^−1^	0.6787	1.0000	0.9264	0
20	Pantothenate and CoA biosynthesis ^b^	1/19	2.58 × 10^−1^	0.5886	1.0000	1.0000	0
21	Ether lipid metabolism ^a^	1/20	2.70 × 10^−1^	0.5694	1.0000	1.0000	0
22	Lysine degradation ^b^	1/25	3.25 × 10^−1^	0.4880	1.0000	1.0000	0
23	Glycolysis/Gluconeogenesis ^b^	1/26	3.36 × 10^−1^	0.4740	1.0000	1.0000	0.0002
24	Glutathione metabolism ^b^	1/28	3.56 × 10^−1^	0.4480	1.0000	1.0000	0.088
25	Porphyrin and chlorophyll metabolism ^b^	1/30	3.77 × 10^−1^	0.4241	1.0000	1.0000	0
26	Inositol phosphate metabolism ^b^	1/30	3.77 × 10^−1^	0.4241	1.0000	1.0000	0.1294
27	Glyoxylate and dicarboxylate metabolism ^b^	1/32	3.96 × 10^−1^	0.4021	1.0000	1.0000	0.1058
28	Amino sugar and nucleotide sugar metabolism ^b^	1/37	4.42 × 10^−1^	0.3541	1.0000	1.0000	0
29	Fatty acid elongation ^b^	1/39	4.60 × 10^−1^	0.3372	1.0000	1.0000	0
30	Fatty acid degradation ^b^	1/39	4.60 × 10^−1^	0.3372	1.0000	1.0000	0
31	Valine, leucine and isoleucine degradation ^b^	1/40	4.69 × 10^−1^	0.3292	1.0000	1.0000	0
32	Fatty acid biosynthesis ^b^	1/47	5.25 × 10^−1^	0.2798	1.0000	1.0000	0.0147
33	Purine metabolism ^b^	1/65	6.45 × 10^−1^	0.1903	1.0000	1.0000	0
34	Biosynthesis of unsaturated fatty acids ^a,b^	6/36	1.02 × 10^−5^	4.9934	0.0009	0.0009	0

Note: ^a^ Discovered in UPLC-Triple-TOF-MS/MS metabolomics analysis; ^b^ Discovered in GC-MS metabolomics analysis; ^a,b^ It was found in both UPLC-Triple-TOF-MS/MS and GC-MS metabolomics analyses.

## Data Availability

The data used to support the findings of this study are available from the corresponding author upon request.

## Supplementary Materials

The following supporting information can be downloaded at: https://www.mdpi.com/article/10.3390/ijms241813695/s1.
