# Supplementary material for: Anti-Rheumatoid Arthritis Pharmacodynamic Substances Screening of Periploca forrestii Schltr.: Component Analyses In Vitro and In Vivo Combined with Multi-Technical Metabolomics
Source: Int J Mol Sci. 2023 Sep 5;24(18):13695. doi: 10.3390/ijms241813695 (PMC10530683; doi:10.3390/ijms241813695)
Supplement: Supplementary file 1 [file ijms-24-13695-s001.zip › ijms-2570449-supplementary.pdf]

Table S1. Component identification *in vitro* and *in vivo* of *P. forrestii* Schltr using UHPLC/Q Exactive Plus high-resolution mass spectrum.

No	Type	Name	Formula	Calc. MW	RT [min]	MS1	MS2	mz Cloud	Confid ence	mz Vault
1	Organic acid	Quinic acid	$C_7H_{12}O_6$	192.06272	1.29	(-)191.05540	(-)173.04471, 155.03459, 127.03892, 111.04396, 93.03327, 85.02821	98.4	97.1	93.2
2 &	Phenolic acid	Protocatechuic acid	$C_7H_6O_4$	154.02572	5.03	(-)153.01840	(-)109.02832	92.1	9.6	99.3
3 &	Phenolic acid	Neochlorogenic acid	$C_{16}H_{18}O_9$	354.09552	7.84	(-)353.08820	(-)191.05554, 179.03432, 135.04414	96.4	63.8	89.9
4	Phenolic aldehyde	Protocatechualde hyde	$C_7H_6O_3$	138.03242	(+)8.16<br>(-)8.17	(+)139.03973<br>(-)137.02350	(+)111.04478, 93.03434;<br>(-)109.02855	(+)73.5<br>(-)95.8	(+)52.9<br>(-)9.8	(+)86.6<br>(-)90.1
5	Coumarin	Esculin	$C_{15}H_{16}O_9$	340.08009	9.77	(-)339.07275	(-)177.01868, 133.02846, 105.03363, 89.03883	98.1	9.9	92.1
6	Alkaloid	Sinomenine	$C_{19}H_{23}NO_4$	329.16399	10.75	(+)330.17130	(+)239.07115, 223.07617, 207.04497, 195.08122, 181.06546, 153.07048, 137.06029	97.9	98.1	88.7
7	Flavanol	Catechin	$C_{15}H_{14}O_6$	290.07964	11.28	(-)289.07227	(-)245.08191, 205.05022, 179.03426, 151.03918, 137.02341	95.1	100	94.6
8 &	Coumarin	Esculetin	$C_9H_6O_4$	178.02720	(+)11.93<br>(-)11.94	(+)179.03439<br>(-)177.01860	(+)151.03946, 133.02885, 123.04458, 105.03421<br>(-)133.02840, 105.03336, 89.03838	(+)98.2	(+)92.4	(+)93.6<br>(-)98.3
9 &	Phenolic acid	Chlorogenic acid	$C_{16}H_{18}O_9$	354.09555	12.45	(-)353.08820	(-)191.05551, 161.02370, 127.03906, 111.04388, 93.03339, 85.02825	99.1	75.8	95.8
10	Organic acid	Caffeic acid	$C_9H_8O_4$	180.04161	12.68	(-)179.03420	(-)135.04407, 117.03342, 107.04914	98.9	9.9	95.1
11	Organic acid	3-Feruloylquinic	$C_{17}H_{20}O_9$	368.11092	13.02	(-)367.10400	(-)193.05006, 134.03630			90

		Acid								
12 &	Phenolic acid	Cryptochlorogenic acid	$C_{16}H_{18}O_9$	354.09508	13.57	(-)353.08826	(-)191.05562, 179.03436, 173.04488, 135.04419	89.6	51.2	94.3
13 &	Coumarin	Fraxin	$C_{16}H_{18}O_{10}$	370.09046	14.68	(-)369.08307	(-)207.02940, 192.00572	98.3	96.6	94.5
14 &	Alkaloid	Magnoflorine	$C_{20}H_{23}NO_4$	341.16444	15.09	(+)342.17178	(+)297.11307, 282.08951, 265.08670, 237.09177			88.5
15 &	Phenolic aldehyde	Vanillin	$C_8H_8O_3$	152.04790	15.13	(+)153.05551	(+)125.06036, 111.04482, 93.03439, 65.03963	95.4	86	90.8
16	Coumarin	Epicatechin	$C_{15}H_{14}O_6$	290.07954	16.13	(-)289.07200	(-)245.08200, 203.07095, 179.03435, 151.03928, 137.02347, 123.04409, 109.02840	95.8	100	94.5
17 &	Coumarin	Fraxetin	$C_{10}H_8O_5$	208.03784	16.28	(+)209.04509	(+)194.02164, 163.03951, 153.05516, 149.02390, 135.04459, 121.02907, 107.04988	98	98.4	94.9
18	Phenolic acid	Ethyl gallate	$C_9H_{10}O_5$	198.05225	17.55	(-)197.04497	(-)168.00545, 140.01039, 125.02325			74.1
19 &	Phenolic acid	Isochlorogenic acid A	$C_{25}H_{24}O_{12}$	516.12770	17.60	(-)515.12042	(-)353.08807, 191.05545, 179.03424, 1353.04408	76.6	67.5	91.9
20	Flavone	Syrangaldehyde	$C_9H_{10}O_4$	182.05867	(+)17.69<br>(-)17.62	(+)183.06595<br>(-)181.05003	(+)140.04710, 123.04449, 95.04984; (-)166.02634, 151.00278, 123.00765			(+)88.3<br>(-)95.4
21	Phenolic acid	Sinapinic acid	$C_{11}H_{12}O_5$	224.06884	19.03	(+)225.07637	(+)207.06573, 175.03944, 147.04443, 119.04971, 91.05500	97.6	96.5	89.0
22	Fatty acid	Jasmonic acid	$C_{12}H_{18}O_3$	210.12347	19.04	(+)211.13077	(+)193.12285, 151.11229, 147.11694, 137.05998, 133.10162	90.8	82.7	
23	Coumarin	Scopoletin	$C_{10}H_8O_4$	192.04305	19.27	(+)193.05042	(+)178.02640, 133.02876	98.6	94.3	93.5

24	Phenolics	Procyanidin A1	$C_{30}H_{24}O_{12}$	576.12778	20.20	(-)575.12006	(-)449.08823, 423.07236, 285.04050, 125.02324			85.3
25	Flavone	Orientin	$C_{21}H_{20}O_{11}$	448.10195	21.30	(-)447.09457	(-)357.06210, 327.05142, 299.05627, 133.02855	97.5	100	90.9
26	Flavone	Piceid	$C_{20}H_{22}O_{8}$	390.13270	21.58	(-)389.12549	(-)227.07104, 185.06003, 143.04924			95.5
27	Phenolic acid	Salicylic acid	$C_{7}H_{6}O_{3}$	138.03078	21.71	(-)137.02350	(-)93.03333, 65.03837	99.4	10	98.8
28	Flavone	Procyanidin A2	$C_{30}H_{24}O_{12}$	576.12797	21.97	(+)577.13647	(+)437.08768, 425.08755, 287.05554, 137.02380			87
29 &	Flavone	Myricitrin	$C_{21}H_{20}O_{12}$	464.09602	21.97	(-)463.08871	(-)316.02261, 287.02002, 271.02505, 214.02669, 178.99792	97.4	100	95.9
30	Phenolic acid	Ferulic acid	$C_{10}H_{10}O_{4}$	194.05791	22.00	(-)193.05020	(-)178.02655, 135.03957, 134.03635	98.0	95.0	93.4
31 &	Flavone	Quercitrin	$C_{21}H_{20}O_{11}$	448.10201	22.04	(+)449.10962	(+)303.05072, 229.05016, 153.01884, 129.05518	97.9	81.7	89.2
32 &	Flavone	Rutin	$C_{27}H_{30}O_{16}$	610.15426	22.07	(+)611.16150	(+)303.05078, 257.04456, 229.05042, 201.05557, 165.01855, 153.01888, 137.02388, 129.05519, 85.02931, 71.05016	99.2	89.6	94.8
33	Phenolic acid	4,5-Diferuloylquinic Acid	$C_{27}H_{28}O_{12}$	544.15920	22.15	(-)543.15192	(-)367.10358, 349.09320, 193.05002, 163.03923, 134.03629			61.6
34 &	Phenolic acid	Isochlorogenic Acid B	$C_{25}H_{24}O_{12}$	516.12750	(+)22.24<br>(-)22.26	(+)499.12531<br>(-)515.12061	(+)319.08200, 163.03938, 145.02890, 135.04453, 117.03413, 89.03941<br>(-)353.08801, 191.05547, 179.03423,	(+)98.8<br>(-)88.4	(+)85.8<br>(-)72.7	(+)93<br>(-)90.8

							173.04474, 1353.04408			
35	Alkaloid	Berberine	$C_{20}H_{17}NO_4$	335.11712	22.28	(+)336.12436	(+)320.09265, 306.07690, 292.09763, 278.08194	96.5	9.8	93.5
36 &	Phenolic acid	Isochlorogenic acid C	$C_{25}H_{24}O_{12}$	516.12739	22.47	(-)515.12006	(-)353.08813, 191.05554, 179.03432, 173.04480, 135.04413	97.4	92.7	91.4
37 &	Polyphenol	Resveratrol	$C_{14}H_{12}O_3$	228.07923	22.54	(+)229.08655	(+)211.07579, 183.08089, 165.07030, 135.04443, 119.04966, 107.04977, 91.05499	99.2	96.3	94.6
38	Flavone	Afzelin	$C_{21}H_{20}O_{10}$	432.10650	22.74	(-)431.09952	(-)285.04047, 255.02994, 227.03474	98.6	70.7	96.6
39	Flavone	Daidzein	$C_{15}H_{10}O_4$	254.05868	22.90	(+)255.06604	(+)224.04762, 209.06090, 197.06017, 180.05731, 135.00769, 133.02850	95.0	9.8	76.6
40	Flavone	Quercetin	$C_{15}H_{10}O_7$	302.04285	22.99	(-)301.03555	(-)273.04065, 245.04573, 178.99800, 151.00284, 121.02845, 107.01275	98.2	94.4	94.9
41 &	Cardiac glycoside	Periplocoside	$C_{36}H_{56}O_{13}$	696.37264	(+)22.78<br>(-)22.80	(+)697.37823<br>(-)695.36658	(+)571.16534, 511.14429, 329.15793, 207.06596<br>(-)452.64120, 353.08826, 191.05559			86.0

Note: & means the component was absorbed into the blood and confirmed using standards.

Table S2. Target genes for 17 blood absorbing components in rats (a total of 574).

ABAT	ABCB1	ABCC1	ABCG2	ABL1	ACE	ACE2	ACHE	ACLY	ACP1
ADA	ADAM17	ADAMTS4	ADAMTS5	ADCY5	ADK	ADORA1	ADORA2A	ADORA2B	ADORA3
ADRA1A	ADRA1B	ADRA1D	ADRA2A	ADRA2B	ADRA2C	ADRB1	ADRB2	AGTR1	AHCY
AHR	AKR1A1	AKR1B1	AKR1B10	AKR1C1	AKR1C2	AKR1C3	AKR1C4	AKT1	ALB
ALDH1A1	ALDH2	ALDH5A1	ALK	ALOX12	ALOX15	ALOX5	ALPG	ALPL	AMD1
AMPD3	AMY2A	ANPEP	AOC3	APEX1	APH1A	APH1B	APP	AR	ASF1A
ATIC	ATP12A	ATP1A1	AURKA	AURKB	AVPR2	AXL	BACE1	BCHE	BCL2
BCL2L1	BMP1	BRAF	BTK	CA1	CA12	CA13	CA14	CA2	CA3
CA4	CA5A	CA5B	CA6	CA7	CA9	CALM1	CAMK2B	CAPN1	CASP1
CASP2	CASP3	CASP6	CASP7	CASP8	CBR1	CCNA1	CCNA1	CCNA2	CCNB1
CCNB2	CCNB3	CCND1	CCND1	CCNE1	CCNE2	CCNT1	CD22	CD38	CDA
CDC25A	CDC25B	CDC7	CDK1	CDK1	CDK2	CDK2	CDK4	CDK5	CDK5R1
CDK9	CDK9	CES1	CFTR	CHEK1	CHEK2	CHRM1	CHRNA4	CHRNA7	CHRNB2
CISD1	CLK1	CLK3	CMA1	CNR1	CNR2	COMT	CSF1R	CSNK1A1	CSNK1D
CSNK2A1	CSNK2A2	CTBP2	CTSB	CTSD	CTSL	CTSV	CXCR1	CXCR2	CYP11B1
CYP11B2	CYP17A1	CYP19A1	CYP1A1	CYP1A2	CYP1B1	CYP2C19	CYP2C9	CYP3A4	DAO
DAPK1	DAPK3	DBF4	DHFR	DHODH	DNM2	DNMT1	DNMT3B	DOT1L	DPP4
DRD1	DRD2	DRD3	DRD4	DRD5	DTYMK	DUSP1	DUSP3	DYRK1A	DYRK1B
DYRK2	ECE1	EDNRA	EGFR	EGLN1	ELANE	ENGASE	ENPEP	EP300	EPHA2
EPHB4	EPHX2	ERAP2	ERBB2	ERN1	ESR1	ESR2	ESRRA	ESRRB	F10
F2	F2RL1	F3	F7	FABP4	FAP	FASN	FBP1	FFAR1	FGFR1
FGR	FLT3	FLT4	FOLH1	FPGS	FTO	FUCA1	FUT7	FYN	GAA
GABRA1	GABRA5	GAPDH	GART	GBA	GBA2	GGH	GLB1	GLI1	GLO1
GLRA1	GLRA2	GNPAT	GPR35	GPR84	GRB2	GRIA2	GRIK2	GRIK3	GRK1

GRK6	GRM2	GRM5	GSK3B	GSR	HCAR2	HCK	HDAC2	HDAC3	HDAC5
HDAC6	HDAC7	HDAC8	HK1	HK2	HMGCR	HMOX1	HPGD	HPRT1	HRAS
HRH2	HSD11B1	HSD11B2	HSD17B1	HSD17B14	HSD17B2	HSD17B3	HSP90AA1	HSP90AB1	HSPA1A
HSPA5	HSPA8	HTR1A	HTR1B	HTR1D	HTR2A	HTR2B	HTR2C	HTR3A	HTR5A
HTR6	HTR7	IARS	ICAM1	ICAM1	IDO1	IGF1R	IGFBP3	IKBKB	IKBKE
IL2	IMPDH1	IMPDH2	INMT	INSR	IRAK4	ITGA2B	ITGA4	ITGA5	ITGAL
ITGAL	ITGAL	ITGAV	ITGB1	ITGB1	ITGB2	ITGB3	ITGB6	ITGB7	JAK3
KAT2B	KCNA3	KCNA5	KCNH2	KCNMA1	KDM2A	KDM3A	KDM4A	KDM4B	KDM4C
KDM4D	KDM4E	KDM5B	KDM5C	KDR	KIF11	KISS1R	KIT	KMT2A	LAP3
LARS	LCK	LCT	LDHA	LDHB	LGALS3	LGALS7	LGALS9	LIMK1	LRRK2
LTB4R	LYN	MAG	MANBA	MAOA	MAOB	MAP2K1	MAP3K14	MAP3K8	MAP3K9
MAPK1	MAPK14	MAPK8	MAPK9	MAPKAPK2	MAPT	MB	MCHR1	MCL1	MET
METAP1	METAP2	MGAM	MGLL	MIF	MKNK1	MME	MMP1	MMP10	MMP12
MMP13	MMP14	MMP16	MMP2	MMP3	MMP7	MMP8	MMP9	MPO	MTOR
MYLK	NAALAD2	NAT1	NCSTN	NEK2	NEU2	NEU3	NEU4	NFKB1	NGFR
NMUR2	NOS1	NOS2	NOS3	NOX4	NPC1L1	NQO1	NQO2	NR3C2	NR4A1
NRP1	NUAK1	OGA	OPRM1	P2RY1	P2RY6	P4HTM	PADI1	PADI2	PADI3
PADI4	PARP1	PDE1B	PDE4B	PDE4D	PDE5A	PDE9A	PDGFRA	PDGFRB	PDPK1
PFKFB3	PGR	PHF8	PI4KB	PIK3CA	PIK3CB	PIK3CD	PIK3CG	PIK3R1	PIM1
PIM3	PIN1	PKN1	PLA2G1B	PLA2G4B	PLAA	PLAT	PLEC	PLG	PLK1
PLK4	PNP	POLA1	POLB	PON1	PPARG	PPP2CA	PPP5C	PREP	PRKACA
PRKCA	PRKCB	PRKCD	PRKCE	PRKCG	PRKCH	PRKCQ	PRKCZ	PRKD1	PRKD2
PRKDC	PRSS1	PSEN1	PSEN2	PSENEN	PSMB5	PTAFR	PTGDR2	PTGES	PTGFR
PTGS1	PTGS2	PTK2	PTK2B	PTPN1	PTPN2	PTPN22	PTPRA	PTPRB	PTPRC
PTPRCAP	PYGL	PYGM	QDPR	RAF1	RARA	RARB	RARG	RBP4	RELA

REN	RET	RNASEH1	RORC	RPS6KA3	RPS6KB1	RXRA	RXRB	RXRG	S1PR1
S1PR3	SELE	SELL	SELP	SERPINE1	SF3B3	SHBG	SI	SIGMAR1	SIRT2
SLC13A5	SLC29A1	SLC37A4	SLC5A1	SLC5A2	SLC6A2	SLC6A3	SLC6A4	SLC8A1	SLC9A1
SMYD2	SNCA	SQLE	SRC	SRD5A1	SRD5A2	STAT3	STS	SUV39H1	SYK
TACR2	TBK1	TBXAS1	TDP1	TEK	TERT	TGM2	TH	TK1	TLR4
TMPRSS15	TNF	TNIK	TNNC1	TNNC1	TNNI3	TNNT2	TNNT2	TOP1	TOP2A
TP53	TPMT	TREH	TRPC3	TRPC6	TRPM8	TRPV1	TSPO	TTL	TTR
TUBB1	TUBB3	TYMP	TYMS	TYR	UGCG	UPP1	VARS	VCAM1	VCP
WEE1	XDH	YARS	YWHAG						

Table S3. Related targets for rheumatoid arthritis (a total of 678).

1C7	6S2650E	ABCG2	ABCG8	ABCP	ABS	ACAN	ACP5	ACSTD1	ACT1
ADAM17	ADAMTS4	ADH1C	ADH3	ADMIO2	AGM1	AGM7	AGS1	AGS2	AGS4
AGXT	AHUS3	AIEFL	AIFEC	AILIM	AILJK	AIS5	AKU	ALB	ALCAM
ALDD	ALPL	ALPS1B	ALPS2	ALPS3	ALPS5	AMPD1	ANGPT1	ANK	ANKH
ANKRD55	ANXA1	AORF	APCS	APO2L	APOH	APT1LG1	ARCND3	ARMD13	ARMD14
ASRT7	ATPLS	B144	BACH2	BACTS2	BCRP	BGLAP	BMP6	BST1	BTNL2
C1QA	C1QB	C1QC	C1QG	C2	C3BR	C4A	C4B	C4BD	C4F
C5orf30	C6	C6orf4	C6orf5	C6orf6	C8B	CACP	CAGA	CAGB	CANDF7
CANDF8	CARD12	CARD14	CARD8	CASP10	CAST	CAT	CCA	CCAL2	CCCKR5
CCL2	CCL20	CCL3	CCL4	CCL5	CCR1	CCR2	CCR5	CCR6	CCR7
CD137	CD150	CD16	CD244	CD247	CD28	CD32	CD4	CD40	CD40LG
CD44	CD64	CD69	CD79A	CD80	CD86	CDC4L	CDCP1	CDKN1A	CDW150
CELIAC1	CELIAC3	CELIAC6	CFAG	CFI	CHI3L1	CIAS1	CIITA	CIP1	CIP2A
CISH	CLAN	CLCNKB	CLDN16	CMDJ	CMKBR2	CMKBR5	CMP	COL11A1	COL2A1

COL3A1	COMP	COPA	CPPDD	CR1	CRISP3	CRP	CRTM	CRV	CSF1
CSF1R	CSF2	CSIF	CTLA4	CTLA8	CTSK	CTSL	CVID1	CVID12	CVID8
CVID9	CXCL1	CXCL10	CXCL12	CXCL2	CXCL5	CXCL6	CXCL8	CXCL9	CXCR3
CXCR4	D6S231E	D6S49E	DDH2	DDX39B	DDX41	DEF6	DEK	DFNA34	DFNA37
DIAR5	DIRA	DMRV	DR3	EDM5	EDN1	EDSCLL1	EDSVASC	EEF1A1	EF1A
ELANE	EOMD	EPCAM	ERAP1	ERG25	F2RL1	FAS	FASL	FASLG	FBN2
FCAS1	FCAS4	FCGR1A	FCGR2A	FCGR2B	FCGR3A	FCGR3B	FCRH3	FCRL3	FCU
FI	FKHRL1	FL1	FLG	FLT1	FN	FN1	FNZ	FOS	FOXO3
FOXO3A	FOXP3	FRP	FRZB	FRZB1	FSTL1	FTDALS3	GALNT3	GBA	GBD4
GCP372	GFI1	GFND2	GOLGB1	GOUT1	GP39	GP5	GPA	GPI	GPR11
GPSM3	GRB1	GURDP	GVHDS	HANK	HAPO	HCP5	HD	HDLCQ7	HERNS
HFE	HFE1	HFE2A	HFTC1	HGD	HHS	HIES4	HJCYS	HJV	HLA-B
HLA-C	HLA-DMA	HLA-DMB	HLA-DOA	HLA-DPB1	HLA-DQA1	HLA-DQA2	HLA-DQB1	HLA-DRA	HLA-DRB1
HLA-DRB5	HLA-G	HLA-H	HMGB1	HNPCC8	HOA	HOMG3	HOPS	HOX4D	HOXD10
HP	HPGD	HRG	HSPA5	HSPD1	HSR	HTT	IBD17	IBD19	IBP
ICAM1	ICOS	IDDM1	IDDM12	IDDM22	IDDM7	IFI1	IFI41	IFI75	IFNB1
IFNG	IGAD1	IGES	IGFR1	IGFR2	IGFR3	IGKC	IGKCD	IL10	IL10RA
IL11	IL12A	IL12B	IL13	IL15	IL17	IL17A	IL17B	IL17RA	IL18
IL19	IL1A	IL1B	IL1R1	IL1RAPL2	IL1RL1	IL1RN	IL2	IL22	IL23A
IL23R	IL2RA	IL2RB	IL3	IL37	IL4	IL6	IL6R	IL6ST	IL7
ILA	ILDR2	IMD20	IMD23	IMD29	IMD31A	IMD31B	IMD31C	IMD36	IMD42
IMD48	IMD57	IMD60	IMD68	IMDSHY	IPAF	IRF1	IRF2	IRF5	IRGM
IT15	ITGA1	ITGAL	ITGAM	ITGB2	ITPR3	JNK2	JUN	KCNJ13	KEFH
KIAA1524	KIAA2025	LACC1	LAF4	LARD	LBA	LCA16	LETS	LMBD1	LMBRD1
LMP2	LMP7	LOMARS	LRBA	LRG47	LST1	LTA	LTB	LYP	M4S1

MACIR	MAFB	MALS	MAN2B2	MAP3K2	MAPK1	MAPK14	MAPK8	MAPK9	MAPKAPK2
MAR	MATN1	MATN3	MBL2	MCCPD	MCH4	MEFV	MEKK2	MERTK	MIC18
MICA	MICB	MIF	MIR132	MIR146A	MIR150	MIR155	MIR198	MK2	MMACHC
MMDD	MMP1	MMP12	MMP13	MMP2	MMP3	MMP8	MMP9	MOM1	MOX2
MPLPF	MPO	MPYS	MSF	MSMO1	MTHFR	MVCD1	MVCD4	MVCD7	MVK
MYD88	MYD88D	NADGP	NAIL	NALP3	NCR3	NESI	NFKB1	NFKBIA	NFKBIL1
NISBD1	NKEFA	NKJO	NKP30	NKR2B4	NKSF2	NLRC4	NLRP1	NLRP12	NLRP3
NOD2	NOS2	NOT	NOTCH2	NR4A2	NRAMP	NRAMP1	NT5	NT5E	NURR1
OATP2A1	OCTN1	OLAH	OPN	OS1	OS2	OSM	P62	p90	PAD
PADI1	PADI2	PADI3	PADI4	PADI5	PAGA	PAR2	PCLN1	PDB3	PDCD1
PDGFRA	PEP	PFITS	PGDH1	PGM3	PGT	PHOAR1	PHOAR2	PIK3CG	PIK3R1
PKDCC	PLA2B	PLA2G2A	PLA2G2F	PLA2G4	PLA2G4A	PLA2L	PLACK	PMGEDSV	PPAC
PPP1CB	PPP1CC	PPRD	PRAAS1	PRAAS3	PRDX1	PRG4	PRKCD	PRKM9	PRTN3
PRXI	PSMB4	PSMB8	PSMB9	PSORS1	PSORS11	PSORS13	PSORS3	PSORS4	PSORS7
PSTPIP1	PTGS1	PTGS2	PTH	PTPN2	PTPN22	PTPN8	PYPAF1	QME	RA
RC3H1	REL	RELB	RETN	RFH1	RING10	RING12	RIP	RIP1	RIPK1
RLSDF	RORC	RORG	RP38	RPEM	RZRG	S100A12	S100A8	S100A9	SAA1
SAA4	SAP	SAVI	SC4MOL	SCN2	SELE	SEMDBCD	SERPINH1	SGK493	SGP28
SHORT	SIAE	SIFD	SIMA135	SLAM	SLAMF1	SLAMF4	SLAT	SLC11A1	SLC19A1
SLC21A2	SLC22A4	SLCO2A1	SLEB11	SLEB2	SMDCF	SP110	SPAT	SPDA1	SPDA3
SPP1	SQSTM1	SRFP3	SRK	SS1	STAT1	STAT3	STAT4	STEAP4	STING1
STL2	STSL1	SVD	SZP	TACE	TAGAP	TEK	TFQTL2	TFRC	TGFB1
TGFB2	TGFB3	THPH11	TIL4	TIMP1	TIMP2	TINUR	TLR2	TLR4	TLR9
TMEM173	TNF	TNFA	TNFAIP3	TNFAIP6	TNFB	TNFBR	TNFR2	TNFRSF11A	TNFRSF11B
TNFRSF12	TNFRSF13C	TNFRSF1A	TNFRSF1B	TNFRSF25	TNFRSF9	TNFSF10	TNFSF11	TNFSF13	TNFSF13B

TNFSF4	TNFSF6	TNIP1	TNSALP	TNX	TNXB	TNXB1	TNXB2	TPMT	TPMTD
TRAF1	TRAF3IP2	TRAIL	TRAT1	TREX1	TRIM	TRNT1	TROP1	TRP1	TRPC1
TTR	UAQTL1	UHS1	UOX	VCAM1	VEGF	VEGFA	VLA1	VLK	VODI
VUR8	WAF1	WDR1	WG	WISP3	YKL40	ZAP70	ZNF163		

Table S4. The core target of *P. forrestii* Schltr. in treating rheumatoid arthritis.

No	Shared name	Betweenness centrality	Closeness centrality	Degree	No	Shared name	Betweenness centrality	Closeness centrality	Degree
1	TNF	0.17134232	0.85714286	40	13	MAPK14	0.01664739	0.62337662	19
2	ALB	0.1114235	0.75	32	14	MMP3	0.00690046	0.61538462	19
3	TLR4	0.08370965	0.75	32	15	MMP1	0.00539825	0.61538462	18
4	STAT3	0.0803477	0.73846154	31	16	NOS2	0.00855764	0.61538462	18
5	MMP9	0.03295401	0.72727273	30	17	SELE	0.00378772	0.60759494	18
6	IL2	0.04871906	0.68571429	27	18	NFKB1	0.00725352	0.6	18
7	VCAM1	0.02228235	0.68571429	26	19	MAPK1	0.01195773	0.58536585	16
8	PTGS2	0.05827588	0.68571429	26	20	ELANE	0.01659297	0.57831325	16
9	ICAM1	0.01126692	0.65753425	24	21	ITGB2	0.00660598	0.56470588	15
10	MPO	0.01664222	0.63157895	21	22	PIK3R1	0.01038103	0.55172414	15
11	MMP2	0.00767392	0.62337662	20	23	CSF1R	0.00927872	0.57142857	14
12	MAPK8	0.01460752	0.62337662	20					

Table S5. Effective component group of *P. forrestii* Schltr. in the treatment of rheumatoid arthritis.

Component	Targets
Protocatechuic acid	TTR/ALB/MMP8/TPMT/ELANE/GBA/TLR4/PTGS1/PTGS2
Neochlorogenic acid	MMP13/MMP2/MMP12/PRKCD/ELANE/MAPK8/PTPN22/ITGAL/PIK3CG/PTGS1/MAPK1/MMP8/MMP1/MMP9/ICAM1/ITGB2
Esculetin	TEK/MIF/NFKB1/MMP9/PTGS2

Chlorogenic acid	CA12/CA9/AKR1B1/CA1/CA14/CA2/GPR35/INSR/CDK2/BACE1/IGF1R/SRC/KDR/GSR/CA5B/MMP9/GRK6/ALOX5
Cryptochlorogenic acid	CA12/CA9/AKR1B1/CA1/CA14/CA2/GPR35/INSR/CDK2/CSNK2A1/BACE1/ESR1/IGF1R/SRC/KDR/GSR/CA5B/CA3/MMP9/GRK6/CLK1/F2/ALOX5/TYMS/DYRK1B
Fraxin	GBA/HSPA5/MAPK1/MMP3/MMP9/MMP1/ADAM17/PTPN2/MMP13/MMP12/MMP8/IL2/TNF/SELE/MMP2
Magnoflorine	MAPK14/MAPKAPK2/CSF1R/HPGD/STAT3/VCAM1/PIK3CG/MIF/MMP9/MMP2/MMP1/PDGFRA/RORC
Vanillin	TTR/MMP1/PTPN22/PIK3CG/VCAM1/TPMT/MPO/MMP9/MMP2/ALPL/MMP8/MMP3/CTSL/MAPK9/MAPK1/ALB
Fraxetin	TEK/MMP9/PIK3CG/ELANE/CTSL/ALPL
Isochlorogenic acid A	MMP13/MMP2/MMP12/ELANE/PRKCD/TTR/MMP1/ADAM17/MAPK1/MMP8/TNF/IL2
Myricitrin	PTGS2/TNF/IL2/ABCG2/PRKCD/MPO/PIK3R1/MMP3/MMP9/PIK3CG
Quercitrin	PTGS2/TNF/IL2/ABCG2/PRKCD/MPO/PIK3R1/MMP3/PIK3CG
Rutin	PTGS2/TNF/IL2/ABCG2/PRKCD/MPO/PIK3R1/MMP13/MMP3/MMP9/PIK3CG/MMP2
Isochlorogenic Acid B	MMP12/MMP2/MMP13/ELANE/TTR/MMP1/SELE/PRKCD/MMP8/ADAMTS4/MMP9/PADI1/PADI4/MAPK8/PADI2/PADI3
Isochlorogenic acid C	MMP12/MMP2/MMP13/ELANE/TTR/MMP1/SELE/PRKCD/MMP8/ADAMTS4/MMP9/PADI1/PADI4/MAPK8/PADI2/PADI3
Resveratrol	PTGS1/PTGS2/TTR/MMP9/MMP1/MMP2/NOS2
Periplocoside	STAT3/RORC/F2RL1/MMP13/GBA/PRKCD/CSF1R/PTGS2/MMP8/ITGAL/MAPK14/ADAMTS4/PTPN22

Table S6. KEGG enrichment results of signaling pathways in the treatment of rheumatoid arthritis with *P. forrestii* Schltr.

Signaling pathways	Count	Log10(P)	Gene names
AGE-RAGE signaling pathway in diabetic complications	13	-14.63	STAT3/PRKCD/MAPK14/MMP2/SELE/MAPK8/TNF/PIK3R1/MAPK1/VCAM1/MAPK9/ICAM1/NFKB1
Lipid and atherosclerosis	16	-14.18	STAT3/MAPK14/MMP9/MMP1/SELE/MAPK8/TNF/PIK3R1/MMP3/HSPA5/MAPK1/VCAM1/MAPK9/ICAM1/TLR4/NFKB1
TNF signaling pathway	13	-13.87	PTGS2/MAPK14/MMP9/SELE/MAPK8/TNF/PIK3R1/MMP3/MAPK1/VCAM1/MAPK9/ICAM1/NFKB1
IL-17 signaling pathway	11	-11.84	MMP13/PTGS2/MAPK14/MMP9/MMP1/MAPK8/TNF/MMP3/MAPK1/MAPK9/NFKB1
C-type lectin receptor signaling pathway	11	-11.35	PRKCD/PTGS2/MAPK14/MAPK8/TNF/IL2/PIK3R1/MAPK1/MAPK9/MAPKAPK2/NFKB1

Fluid shear stress and atherosclerosis	12	-11.32	MAPK14/MMP9/MMP2/SELE/MAPK8/TNF/PIK3R1/CTSL/VCAM1/MAPK9/ICAM1/NFKB1
Relaxin signaling pathway	11	-10.32	MMP13/MAPK14/MMP9/MMP1/MMP2/NOS2/MAPK8/PIK3R1/MAPK1/MAPK9/NFKB1
Chagas disease	10	-10.00	MAPK14/NOS2/MAPK8/TNF/IL2/PIK3R1/MAPK1/MAPK9/TLR4/NFKB1
Coronavirus disease - COVID-19	13	-9.88	STAT3/MAPK14/MMP1/MAPK8/TNF/IL2/PIK3R1/MMP3/MAPK1/ADAM17/MAPK9/TLR4/NFKB1
Pertussis	9	-9.76	MAPK14/NOS2/MAPK8/TNF/MAPK1/MAPK9/ITGB2/TLR4/NFKB1
Toxoplasmosis	10	-9.59	STAT3/MAPK14/NOS2/MAPK8/TNF/PIK3CG/MAPK1/MAPK9/TLR4/NFKB1
Rheumatoid arthritis	9	-8.96	ITGAL/MMP1/TNF/MMP3/TEK/CTSL/ICAM1/ITGB2/TLR4
Kaposi sarcoma-associated herpesvirus infection	11	-8.41	STAT3/PTGS2/MAPK14/MAPK8/PIK3R1/PIK3CG/MAPK1/MAPK9/MAPKAPK2/ICAM1/NFKB1
Leishmaniasis	8	-8.24	PTGS2/MAPK14/NOS2/TNF/MAPK1/ITGB2/TLR4/NFKB1
Malaria	7	-8.18	ITGAL/SELE/TNF/VCAM1/ICAM1/ITGB2/TLR4
Hepatitis B	10	-8.01	STAT3/MAPK14/MMP9/MAPK8/TNF/PIK3R1/MAPK1/MAPK9/TLR4/NFKB1
Yersinia infection	9	-7.47	MAPK14/MAPK8/TNF/IL2/PIK3R1/MAPK1/MAPK9/TLR4/NFKB1
Neutrophil extracellular trap formation	10	-7.35	ITGAL/MAPK14/ELANE/PADI4/MPO/PIK3R1/MAPK1/ITGB2/TLR4/NFKB1
Toll-like receptor signaling pathway	8	-7.20	MAPK14/MAPK8/TNF/PIK3R1/MAPK1/MAPK9/TLR4/NFKB1
T cell receptor signaling pathway	8	-7.20	MAPK14/MAPK8/TNF/IL2/PIK3R1/MAPK1/MAPK9/NFKB1

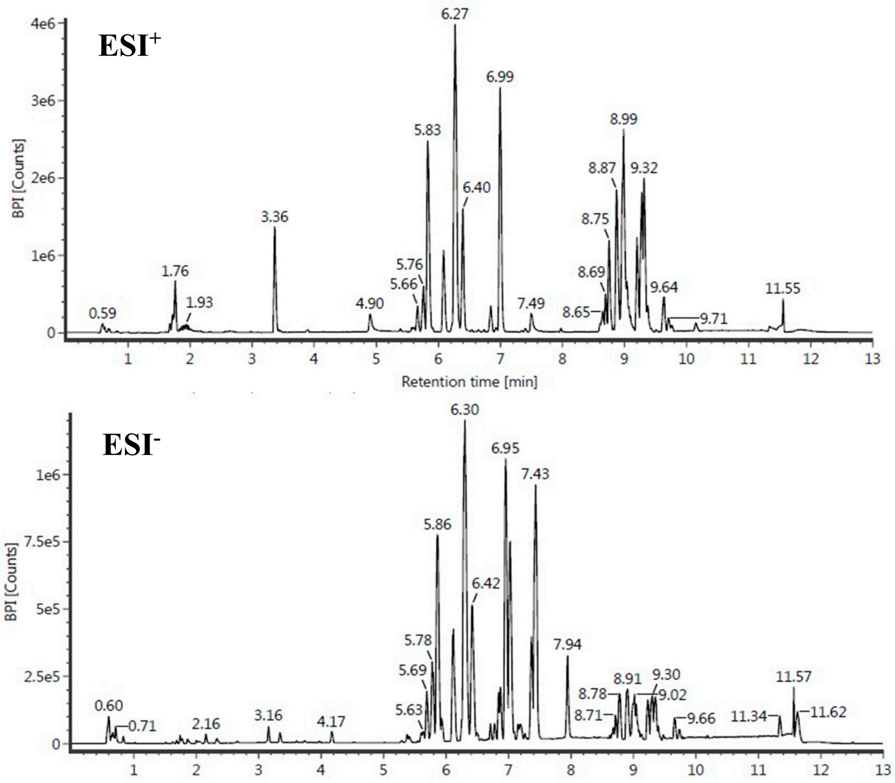

Figure S1. Total ion chromatogram of QC plasma samples detected by LC-MS metabolomics.

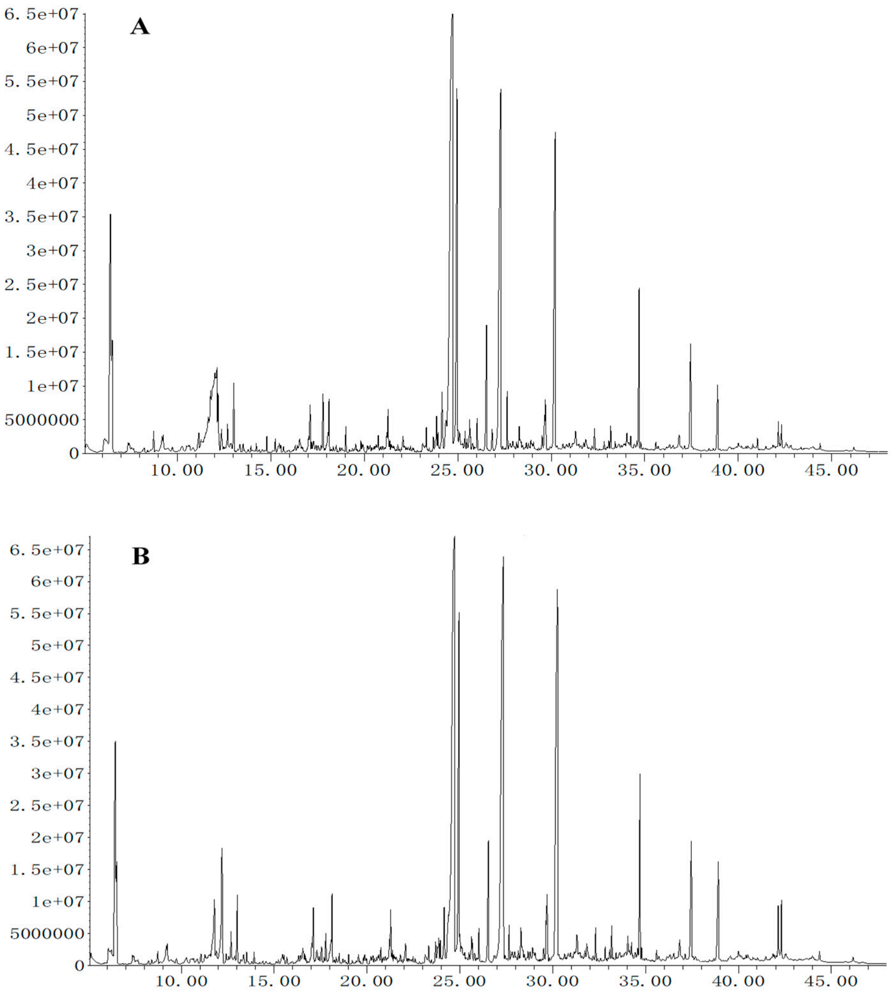

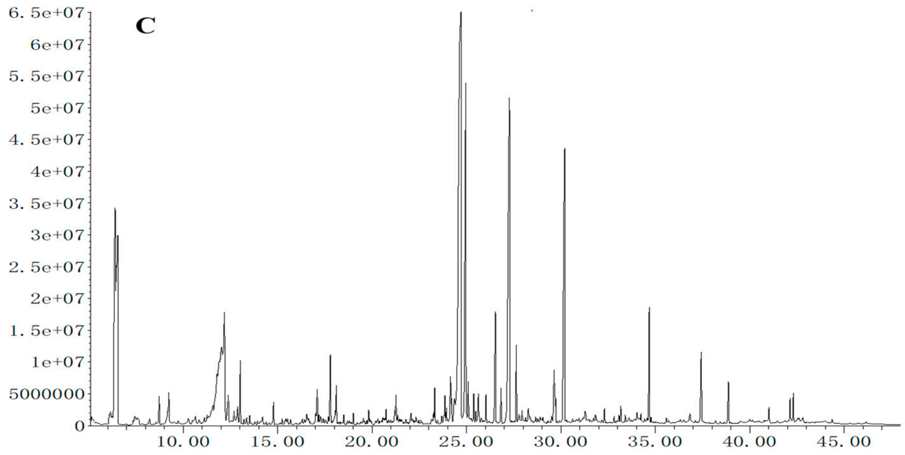

Figure S2. GC-MS metabolomics detection of total ion chromatogram in plasma samples; (A) CG, (B) MG, and (C) TG.

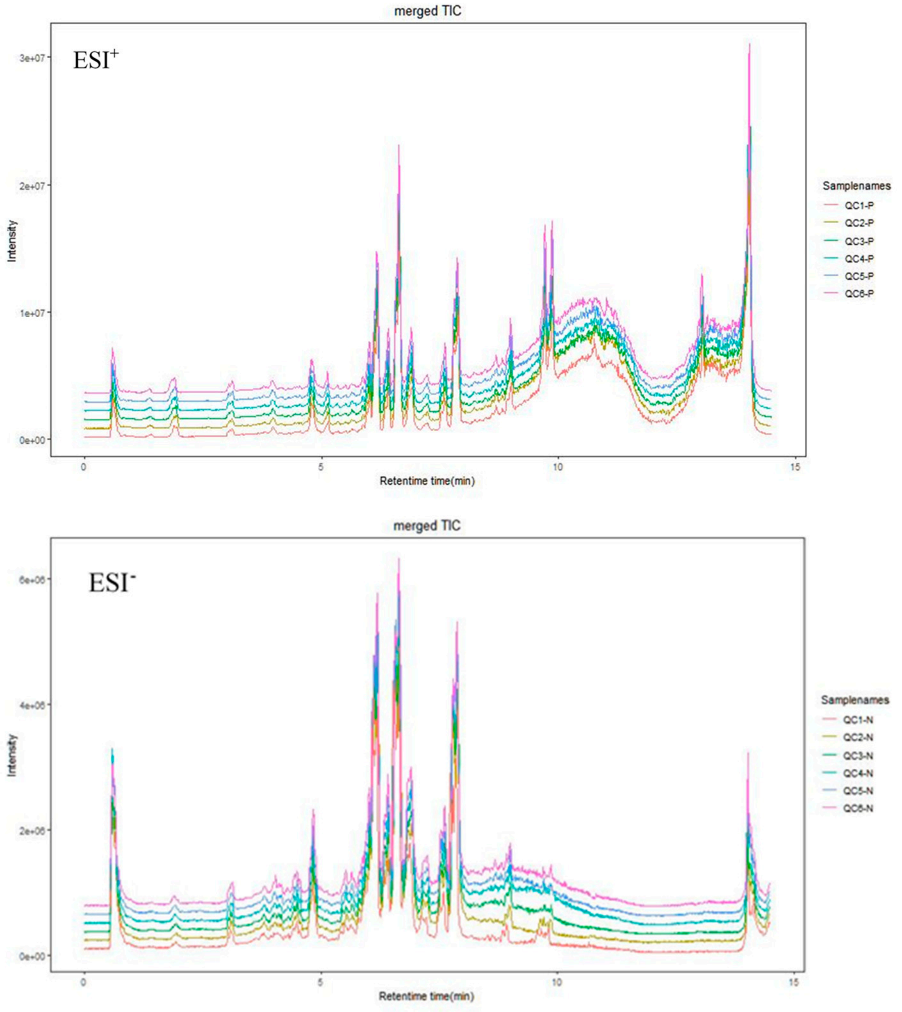

Figure S3. LC-MS system reproducibility study for analysis protocol based on TIC of QC plasma samples (n=6)

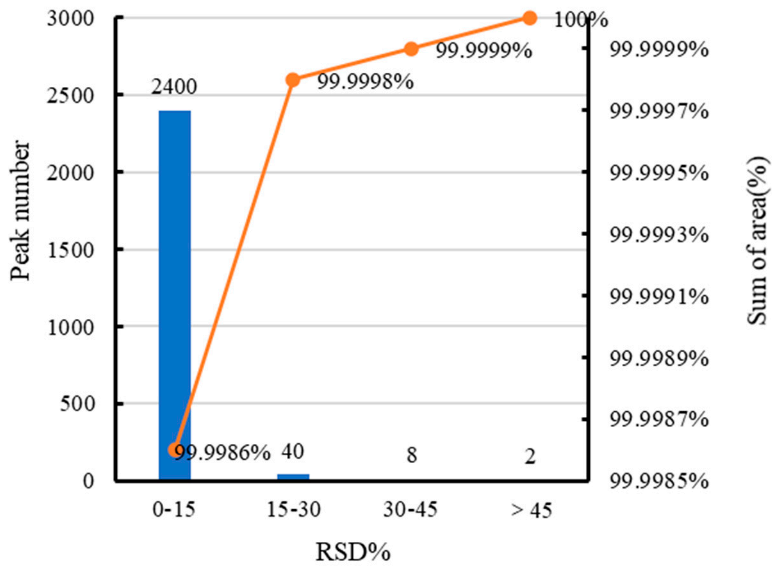

Figure S4. Repeatability of the metabolomics study using UPLC-Triple-TOF-MS/MS . RSD distribution plot of all metabolites in QC samples were calculated. The column represents the peak number within the specific RSD range, and the line represents the percentage of cumulative peak area within the specific RSD range, respectively.

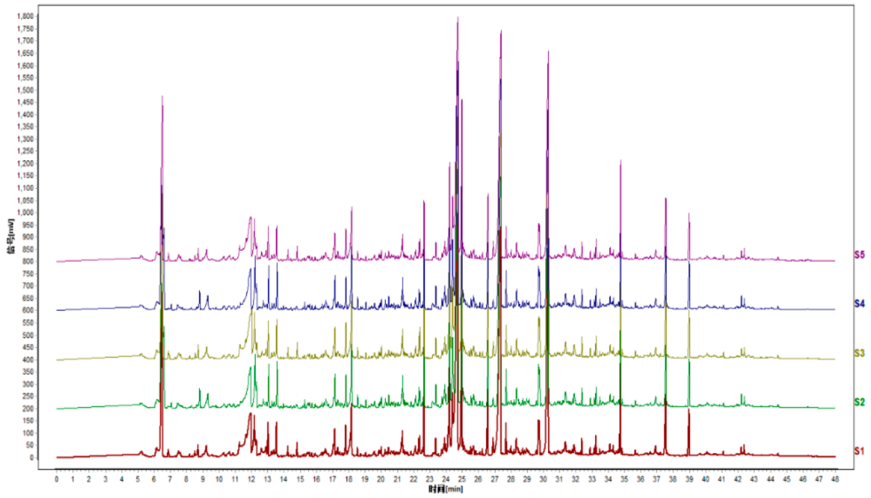

(**a**)

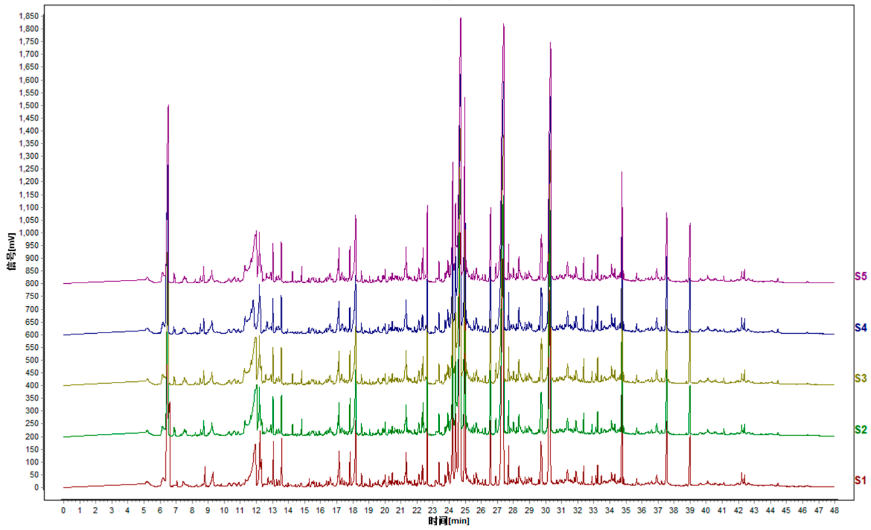

(**b**)

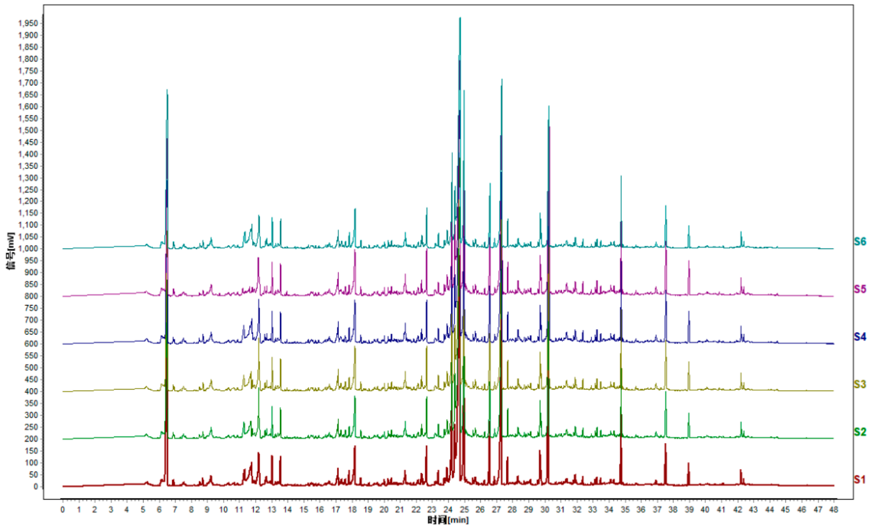

Note: S1 to S6 are the GC-MS results at 0, 2, 6, 12, 18, and 24 hours, respectively

(**c**)

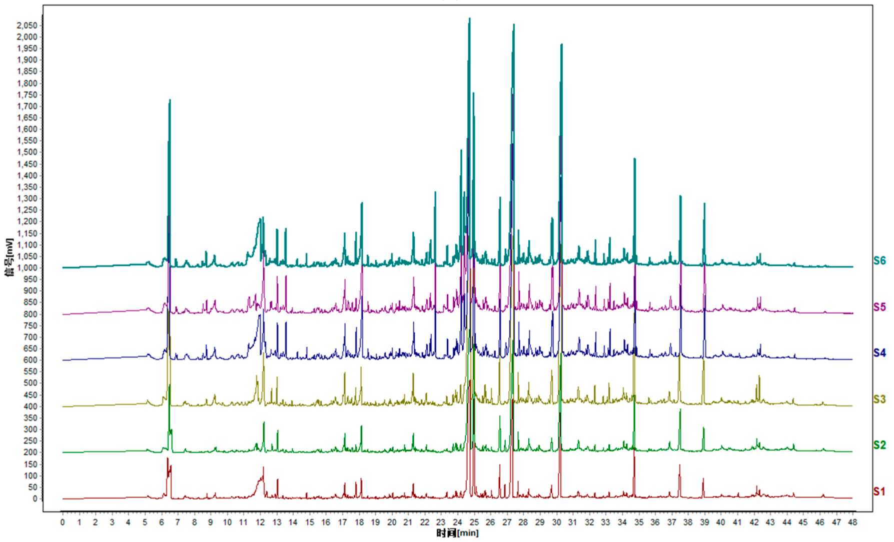

(**d**)

Figure.S5 GC-MS system reproducibility study for analysis protocol based on TIC of QC plasma samples (*n*=6): (a) Precision, (b) Reproducibility, (c) Stability, (d) Stability of a system.

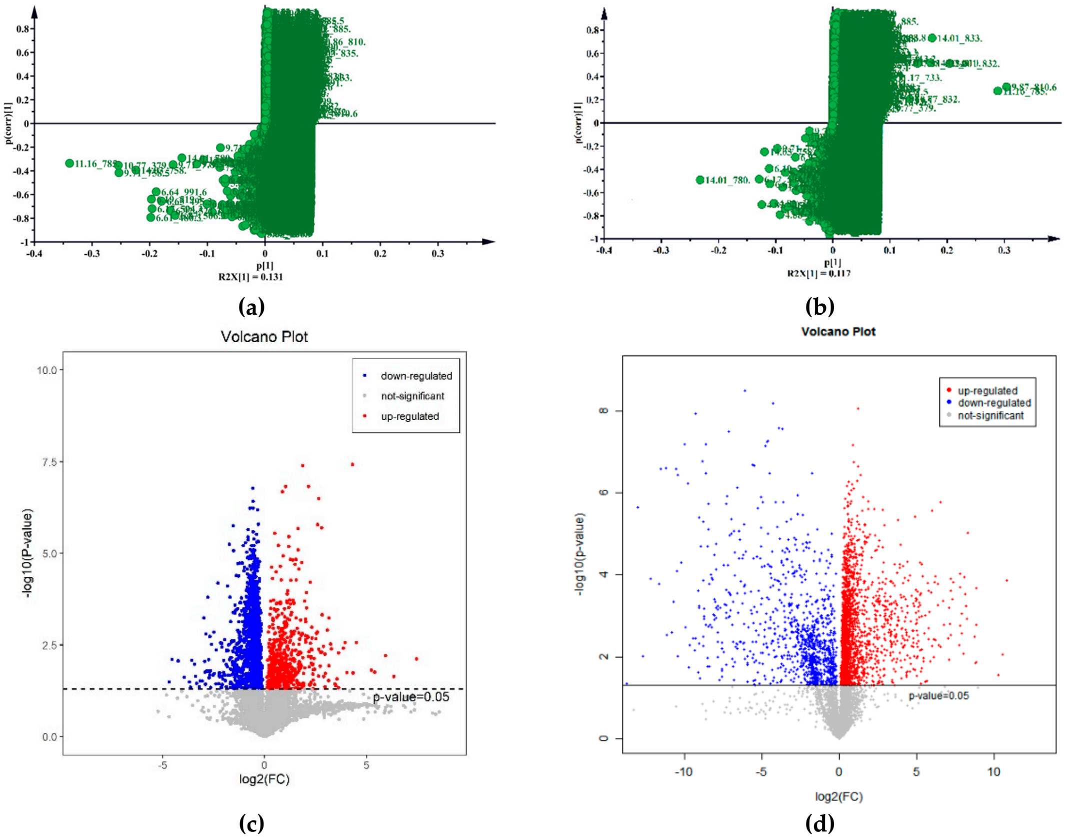

Figure S6. Screening of differential metabolites using s-plot and volcano plots of plasma samples between different groups: taking UPLC Triple TOF-MS/MS analysis results as an example. s-Plot of OPLS-DA analysis(**a, b**) ; Volcano plot of OPLS-DA analysis in CG-MG and MG-TG for UPLC-Triple-TOF-MS/MS (**c, d**).

Table S7. Significantly changed plasma metabolites associated with RA were detected by UPLC-Triple-TOF-MS and GC-MS.

No	Metabolites	m/z	RT (min)	Compound ID	Formula	Tend	Metabolic pathway
LC 1	Arachidonic acid	303.23	6.14	HMDB0001043	$C_{20}H_{32}O_2$	↓	Arachidonic acid metabolism
LC 2	DG(18:0/20:4(5Z,8Z,11Z,14Z)/0:0)	627.53	14.06	HMDB0007170	$C_{41}H_{72}O_5$	↓	Glycerolipid metabolism
LC 3	Chenodeoxycholic Acid	393.3	5.52	207	$C_{24}H_{40}O_4$	↓	Primary bile acid biosynthesis
LC 4	Deoxycorticosterone	678.47	9.53	HMDB0000016	$C_{21}H_{30}O_3$	↓	Steroid hormone biosynthesis
LC 5	norcisapride	625.23	6.55	HMDB0061018	$C_{14}H_{20}ClN_3O_3$	↓	unknown
LC 6	LysoPA(0:0/16:0)	409.24	6.62	HMDB0007849	$C_{19}H_{39}O_7P$	↓	Glycerophospholipid metabolism
LC 7	Leukotriene C5	646.28	7.78	HMDB0012993	$C_{30}H_{45}N_3O_9S$	↓	Arachidonic acid metabolism
LC 8	D-Lactose	341.11	3.15	267	$C_{12}H_{22}O_{11}$	↓	Carbohydrate digestion and absorption
LC 9	Xeniasterol-a	493.39	12.5	LMST01031064	$C_{30}H_{52}O_5$	↓	unknown
LC 10	PS(12:0/15:0)	648.42	13.89	LMGP03010047	$C_{33}H_{64}NO_{10}P$	↓	Glycerophospholipid metabolism
LC 11	LysoPE(0:0/20:3(11Z,14Z,17Z))	502.29	5.74	HMDB0011484	$C_{25}H_{46}NO_7P$	↓	Glycerophospholipid metabolism
LC 12	PG(18:3(6Z,9Z,12Z)/0:0)	507.27	8.09	LMGP04050020	$C_{24}H_{43}O_9P$	↓	Glycerophospholipid metabolism
LC 13	Glycerophosphocholine	258.11	6.19	370	$C_8H_{20}NO_6P$	↓	Glycerophospholipid metabolism
LC 14	Isopalmitic acid	255.23	6.61	4289	$C_{16}H_{32}O_2$	↓	Biosynthesis of unsaturated fatty acids
LC 15	LysoPC(18:1(9Z))	566.35	6.87	HMDB0002815	$C_{26}H_{52}NO_7P$	↓	Glycerophospholipid metabolism
LC 16	Choline	104.11	6.92	56	$C_5H_{13}NO$	↓	Glycine, serine, and threonine metabolism
LC 17	Iso-Valeraldehyde	104.11	6.19	HMDB0006478	$C_5H_{10}O$	↓	Glycine, serine, and threonine metabolism
LC 18	Tryptophanol	184.07	6.02	HMDB0003447	$C_{10}H_{11}NO$	↓	Tryptophan metabolism
LC 19	Phosphocholine	184.07	6.19	3318	$C_5H_{14}NO_4P$	↓	Glycerophospholipid metabolism
LC 20	Indoleacrylic acid	188.07	1.87	HMDB0000734	$C_{11}H_9NO_2$	↓	Linoleic acid metabolism
LC 21	10E,12Z-Octadecadienoic acid	279.23	6.17	34801	$C_{18}H_{32}O_2$	↑	Linoleic acid metabolism
LC 22	LysoPC(18:2(9Z,12Z))	520.34	6.19	HMDB0010386	$C_{26}H_{50}NO_7P$	↓	Glycerophospholipid metabolism
LC 23	Linoleic acid	279.23	8.9	HMDB0000673	$C_{18}H_{32}O_2$	↑	alpha-Linolenic acid metabolism
LC 24	8,11-eicosadiynoic acid	303.23	8.72	24087	$C_{20}H_{32}O_2$	↑	Linoleic acid metabolism
LC 25	PE(20:2(11Z,14Z)/22:4(7Z,10Z,13Z,16Z))	818.57	14.00	HMDB0009306	$C_{47}H_{82}NO_8P$	↑	Glycerophospholipid metabolism
LC 26	PG(a-13:0/a-13:0)	677.38	13.03	HMDB0116636	$C_{32}H_{63}O_{10}P$	↑	Glycerophospholipid metabolism
LC 27	PC(15:0/20:0)	814.57	14.01	HMDB0007944	$C_{43}H_{86}NO_8P$	↑	Glycerophospholipid metabolism
LC 28	PC(22:4(7Z,10Z,13Z,16Z)/P-18:1(11Z))	842.6	13.99	HMDB0008654	$C_{48}H_{86}NO_7P$	↑	Glycerophospholipid metabolism
LC 29	PI(18:0/20:4(5Z,8Z,11Z,14Z))	885.55	14.01	HMDB0009815	$C_{47}H_{83}O_{13}P$	↑	Glycerophospholipid metabolism

No	Metabolites	m/z	RT (min)	Compound ID	Formula	Tend	Metabolic pathway
LC 30	PI(16:0/20:4(5Z,8Z,11Z,14Z))	857.52	7.64	HMDB0009789	$C_{45}H_{79}O_{13}P$	↑	Glycerophospholipid metabolism
LC 31	PE(15:0/22:2(13Z,16Z))	802.56	14.01	HMDB0008909	$C_{42}H_{80}NO_8P$	↑	Glycerophospholipid metabolism
LC 32	Glucosylceramide (d18:1/16:0)	722.55	12.6	HMDB0004971	$C_{40}H_{77}NO_8$	↑	Sphingolipid metabolism
LC 33	SM(d18:0/24:1(15Z)(OH))	811.67	14.03	HMDB0013469	$C_{47}H_{93}N_2O_7P$	↑	Sphingolipid metabolism
LC 34	Lactosylceramide(d18:1/16:0)	844.62	11.06	HMDB0006750	$C_{46}H_{87}NO_{13}$	↑	Sphingolipid metabolism
GC 1	Propanoic acid	115.0	6.69	HMDB0000237	$C_3H_6O_2$	↑	Propanoate metabolism
GC 2	(R)-3-Hydroxybutyric acid	73.1	9.49	HMDB0000011	$C_4H_8O_3$	↑	Synthesis and degradation of ketone bodies
GC 3	Estragole	148.1	10.74	HMDB0034121	$C_{10}H_{12}O$	↑	unknown
GC 4	L-Valine	73.1	10.85	HMDB0000883	$C_5H_{11}NO_2$	↑	Valine,leucine and isoleucine biosynthesis
GC 5	Butanoic acid	85.1	11.57	HMDB0000039	$C_4H_8O_2$	↓	Butanoate metabolism
GC 6	Urea	71.1	12.22	HMDB0000294	CH_4N_2O	↓	Purine metabolism
GC 7	Glycerol	131.1	12.36	HMDB0000131	$C_3H_8O_3$	↑	Glycerolipid metabolism
GC 8	Aminoethane sulfonic acid	91.1	12.56	HMDB0000251	H_3NO_3S	↓	Taurine and hypotaurine metabolism
GC 9	Glycine	57.1	13.10	HMDB0000123	$C_2H_5NO_2$	↓	Glycine, serine and threonine metabolism
GC 10	Butane dioic acid	221.1	13.47	HMDB0000254	$C_4H_6O_4$	↓	Citrate cycle (TCA cycle)
GC 11	1,3,5-Triazine	191.1	19.69	NA	$C_3H_3N_3$	↓	unknown
GC 12	L-phenylalanine	43.1	20.08	HMDB0000159	$C_9H_{11}NO_2$	↓	Phenylalanine metabolism
GC 13	D-Ribose	217.1	20.38	HMDB0000283	$C_5H_{10}O_5$	↑	Pentose phosphate pathway
GC 14	D-(+)-Xylose	217.1	20.95	HMDB0000098	$C_5H_{10}O_5$	↑	Pentose and glucuronate interconversions
GC 15	Hentriacontane	111.1	21.48	HMDB0030092	$C_{31}H_{64}$	↓	Unknown
GC 16	DL-Ornithine	142.1	23.47	HMDB32455	$C_5H_{12}N_2O_2$	↑	Unknown
GC 17	1,5-Anhydro-D-sorbitol	258.2	24.04	HMDB0002712	$C_6H_{12}O_5$	↑	Unknown
GC 18	D-Fructose	70.1	24.36	HMDB0000660	$C_6H_{12}O_6$	↓	Galactose metabolism
GC 19	d-Mannose	220.1	24.9	HMDB0000169	$C_6H_{12}O_6$	↑	Fructose and mannose metabolism
GC 20	d-Glucose	113.1	25.2	HMDB0000122	$C_6H_{12}O_6$	↓	Starch and sucrose metabolism
GC 21	L-Lysine	56.1	25.28	HMDB0000182	$C_6H_{14}N_2O_2$	↓	Lysine degradation
GC 22	Myo-Inositol	265.1	27.86	HMDB0000211	$C_6H_{12}O_6$	↓	Inositol phosphate metabolism
GC 23	Heptacosane	57.1	29.15	NA	$C_{27}H_{56}$	↓	Unknown
GC 24	9,12-Octadecadienoic acid	144.1	29.90	HMDB0000673	$C_{18}H_{32}O_2$	↑	Linoleic acid metabolism
GC 25	Oleic acid	160.1	29.91	HMDB0000207	$C_{18}H_{34}O_2$	↑	Biosynthesis of unsaturated fatty acids
GC 26	Arachidonic acid	41.1	32.08	HMDB0001043	$C_{20}H_{32}O_2$	↓	Arachidonic acid metabolism

No	Metabolites	m/z	RT (min)	Compound ID	Formula	Tend	Metabolic pathway
GC 27	Eicosanoic acid	369.4	33.06	HMDB0002212	$C_{20}H_{40}O_2$	↑	Biosynthesis of unsaturated fatty acids
GC 28	Hexadecanoic acid	70.1	34.91	HMDB0000220	$C_{16}H_{32}O_2$	↓	Biosynthesis of unsaturated fatty acids
GC 29	Tetracosane	43.1	36.60	NA	$C_{24}H_{50}$	↓	Unknown
GC 30	Octadecanoic acid	97.1	37.79	HMDB0000827	$C_{18}H_{36}O_2$	↑	Biosynthesis of unsaturated fatty acids
GC 31	Octadecane	99.1	38.22	HMDB0033721	$C_{18}H_{38}$	↓	Unknown
GC 32	Triacontane	113.1	40.94	NA	$C_{30}H_{62}$	↓	Unknown

* " ↓ " or " ↑ " means the metabolite significantly decreased or increased in AA Model group compared with control group.

Table S8. Main metabolic pathways related to RA according to MS analyses and MetaboAnalyst database.

No	Pathways	Total	Expected	Hits	Raw p	Holm adjust	FDR	Impact	Dctails
LC 1	Glycerophospholipid metabolism	36	0.4413	7	8.19E-08	6.88E-06	6.88E-06	0.4395	KEGG
LC 2	Linoleic acid metabolism	5	0.0613	2	0.0014	0.1157	0.0506	1	KEGG
LC 3	Sphingolipid metabolism	21	0.2574	3	0.0018	0.1483	0.0506	0.0385	KEGG
LC 4	Biosynthesis of unsaturated fatty acids	36	0.4413	2	0.0700	1	1	0	KEGG
LC 5	Arachidonic acid metabolism	36	0.4413	2	0.0700	1	1	0.3135	KEGG
LC 6	alpha-Linolenic acid metabolism	13	0.1594	1	0.1487	1	1	0	KEGG
LC 7	Glycosylphosphatidylinositol (GPI)-anchor biosynthesis	14	0.1716	1	0.1592	1	1	0.0040	KEGG
LC 8	Glycerolipid metabolism	16	0.1961	1	0.1799	1	1	0.0125	KEGG
LC 9	Ether lipid metabolism	20	0.2452	1	0.2198	1	1	0	KEGG
LC 10	Phosphatidylinositol signaling system	28	0.3432	1	0.2942	1	1	0.0015	KEGG
LC 11	Glycine, serine and threonine metabolism	33	0.4045	1	0.3372	1	1	0	KEGG
LC 12	Primary bile acid biosynthesis	46	0.5639	1	0.4378	1	1	0	KEGG
LC 13	Steroid hormone biosynthesis	85	1.0419	1	0.6597	1	1	0.0221	KEGG
GC 1	Linoleic acid metabolism	5	0.0871	1	0.0842	1	0.8843	1	KEGG
GC 2	Phenylalanine,tyrosine and tryptophan biosynthesis	4	0.0697	1	0.0679	1	0.8843	0.5	KEGG
GC 3	Taurine and hypotaurine metabolism	8	0.1394	1	0.1314	1	1	0.4286	KEGG
GC 4	Phenylalanine metabolism	10	0.1742	1	0.1616	1	1	0.3571	KEGG
GC 5	Arachidonic acid metabolism	36	0.6271	1	0.4727	1	1	0.3135	KEGG
GC 6	Glycine, serine, and threonine metabolism	33	0.5748	1	0.4435	1	1	0.2458	KEGG
GC 7	Glycerolipid metabolism	16	0.2787	1	0.2461	1	1	0.2368	KEGG
GC 8	Inositol phosphate metabolism	30	0.5226	1	0.4127	1	1	0.1294	KEGG
GC 9	Glyoxylate and dicarboxylate metabolism	32	0.5574	1	0.4334	1	1	0.1058	KEGG
GC 10	Glutathione metabolism	28	0.4877	1	0.3913	1	1	0.0887	KEGG
GC 11	Pentose and glucuronate interconversions	18	0.3136	1	0.2725	1	1	0.0781	KEGG
GC 12	Phosphatidylinositol signaling system	28	0.4877	1	0.3913	1	1	0.0374	KEGG
GC 13	Citrate cycle (TCA cycle)	20	0.3484	1	0.2979	1	1	0.0327	KEGG
GC 14	Primary bile acid biosynthesis	46	0.8013	2	0.1898	1	1	0.0152	KEGG
GC 15	Fatty acid biosynthesis	47	0.8187	1	0.5677	1	1	0.0147	KEGG
GC 16	Glycolysis/Gluconeogenesis	26	0.4529	1	0.3691	1	1	0.0102	KEGG

Note: The “total” is the total number of compounds in the pathway; The “hits” is the actual number of matches in the user's uploaded data; “Raw p” is the original p-value calculated through enrichment analysis; “Holm adjust” is the p-value adjusted through the Holm Bonferroni method; “FDR” is the error detection rate; “Impact” is the path impact value calculated based on path topology analysis.
